# Knockoff-ML: a knockoff machine learning framework for controlled variable selection and risk stratification in electronic health record data

**DOI:** 10.1038/s41746-025-02102-2

**Published:** 2025-11-26

**Authors:** Qi Wang, Linyan Li, Yi Yang

**Affiliations:** 1 Department of Data Science, City University of Hong Kong, Kowloon, Hong Kong SAR; 2 Department of Infectious Diseases and Public Health, City University of Hong Kong, Kowloon, Hong Kong SAR; 3 Department of Biostatistics, City University of Hong Kong, Kowloon, Hong Kong SAR

**Keywords:** Computational biology and bioinformatics, Health care, Mathematics and computing, Medical research

## Abstract

Effective risk stratification is essential in clinical practice, enabling better resource allocation and improved patient outcomes. Although machine learning models have been widely used for risk prediction and stratification in electronic health record (EHR) data, conventional interpretability metrics for machine learning models typically lack decision rules for clinicians to determine which patient features significantly influence outcomes. We proposed Knockoff-ML, a model-free machine learning framework that simultaneously accomplishes outcome prediction and identification of risk features through integrating a knockoff framework with various predictive machine learning algorithms. Specifically, Knockoff-ML augments traditional machine learning models with the knockoff framework that enables machine learning models to perform variable selection with false discovery rate (FDR) control in the presence of complex, nonlinear associations between features and outcomes in EHR data. We extensively evaluated Knockoff-ML in both simulations and real-data applications. Our simulation results demonstrated that Knockoff-ML consistently achieved high statistical power to identify risk features while rigorously controlling the FDR, whereas conventional feature selection methods exhibited inflated FDR in most scenarios. In applications to a cohort of 50,591 intensive care unit (ICU) patients from the Medical Information Mart for Intensive Care (MIMIC)-IV database, Knockoff-ML identified risk features significantly associated with short- and long-term mortality. Prediction models with identified risk features in Knockoff-ML also achieved comparable prediction accuracy with full models using all available features. Furthermore, Knockoff-ML exhibited substantially higher predictive power and clinical utility compared to conventional ICU scoring systems such as SOFA and SAPS II. The robust performance and interpretability of Knockoff-ML make it a useful tool for enhancing clinical decision-making, with the potential to significantly improve patient outcomes and optimize healthcare delivery.

## Introduction

Risk stratification is a cornerstone of clinical practice, enabling healthcare providers to categorize patients based on their likelihood of experiencing adverse outcomes^1,2^. By evaluating risk factors such as age, laboratory results, and medical history, clinicians can identify individuals at higher risk of complications or those who may require intensive care^3^. This process not only facilitates better resource allocation but also improves patient outcomes, ultimately contributing to more efficient and effective healthcare delivery^4^.

To support risk stratification, several severity scoring systems have been developed, including the simplified acute physiology score II (SAPS II)^5^, acute physiology and chronic health evaluation (APACHE)^6^, and sequential organ failure assessment (SOFA)^7^. These traditional scoring systems, grounded in clinical expertise, assess physiological parameters and organ dysfunction to provide valuable insights into patient risk^8^. Recently, with the rapid advancement of machine learning (ML) techniques, there has been a paradigm shift toward data-driven approaches for risk prediction. ML models, such as random forests (RF)^9^, extreme gradient boosting (XGBoost)^10^, and categorical boosting (CatBoost)^11^, have demonstrated superior precision in predicting adverse outcomes and assessing critical illness risk. These ML models exhibit a markedly improved capability to incorporate a wider range of predictors^12^ and effectively address nonlinearities^13^ within clinical applications.

Despite the advantages in outcome prediction, ML methods often operate as “black-box” models, lacking interpretability on which patient features are significantly associated with outcomes. To address this limitation, interpretability metrics such as Shapley additive explanation (SHAP)^14,15^ values have been proposed to measure feature importance in ML models by quantifying the contribution of each feature to model predictions. However, determining the optimal number of variables to select as significant features based on SHAP values remains a subjective decision. Consequently, both conventional feature selection methods and ML-based feature importance approaches share a common limitation: the absence of a robust, objective criterion for variable selection with statistical rigor that guarantees a high proportion of selected variables are truly significant features.

In this study, we introduce Knockoff-ML, a knockoff-augmented machine learning framework that simultaneously achieves variable selection and risk prediction in electronic health record (EHR) data. The knockoff framework controls the false discovery rate (FDR)^16–18^, defined as the proportion of irrelevant features among all selected features. By generating synthetic “knockoff” variables that mimic the correlation structure of the original features, the framework rigorously tests the significance of each variable in the presence of complex correlation among variables. Features are significant only if they outperform their knockoff counterparts considerably in feature importance (e.g., SHAP values) based on a significance threshold determined by target FDR levels. Therefore, Knockoff-ML enables ML models to perform FDR-informed variable selection that statistically guarantees a high proportion of selected variables are truly risk features. The selected features are further used for risk prediction in ML models. This mitigates the risk of overfitting in ML models compared to using all available features, making it particularly well-suited for clinical datasets with limited sample sizes.

Specifically, Knockoff-ML consists of four main components: (i) generating knockoff features, (ii) fitting ML models and calculating feature importance (e.g., SHAP values), (iii) performing knockoff-based variable selection to identify significant features with FDR control, and (iv) training ML models with identified features for risk prediction. We validated Knockoff-ML through simulation experiments and demonstrated its application in variable selection and mortality risk prediction in intensive care units (ICU) using the Medical Information Mart for Intensive Care (MIMIC)-IV^19^ dataset. Knockoff-ML identified risk features significantly associated with 7-day, 30-day, and 1-year mortality, revealing that these features differentially impact short-term and long-term mortality. Meanwhile, Knockoff-ML with identified risk features achieved competitive prediction performance compared to models trained with all features, and significantly outperformed conventional scoring systems such as SOFA^7^ and SAPS II^5^. Knockoff-ML advances the field of risk stratification, offering a robust, interpretable, and scalable solution for controlled variable selection and improved risk prediction in clinical practice.

## Results

### Overview of Knockoff-ML

**Fig. 1:**
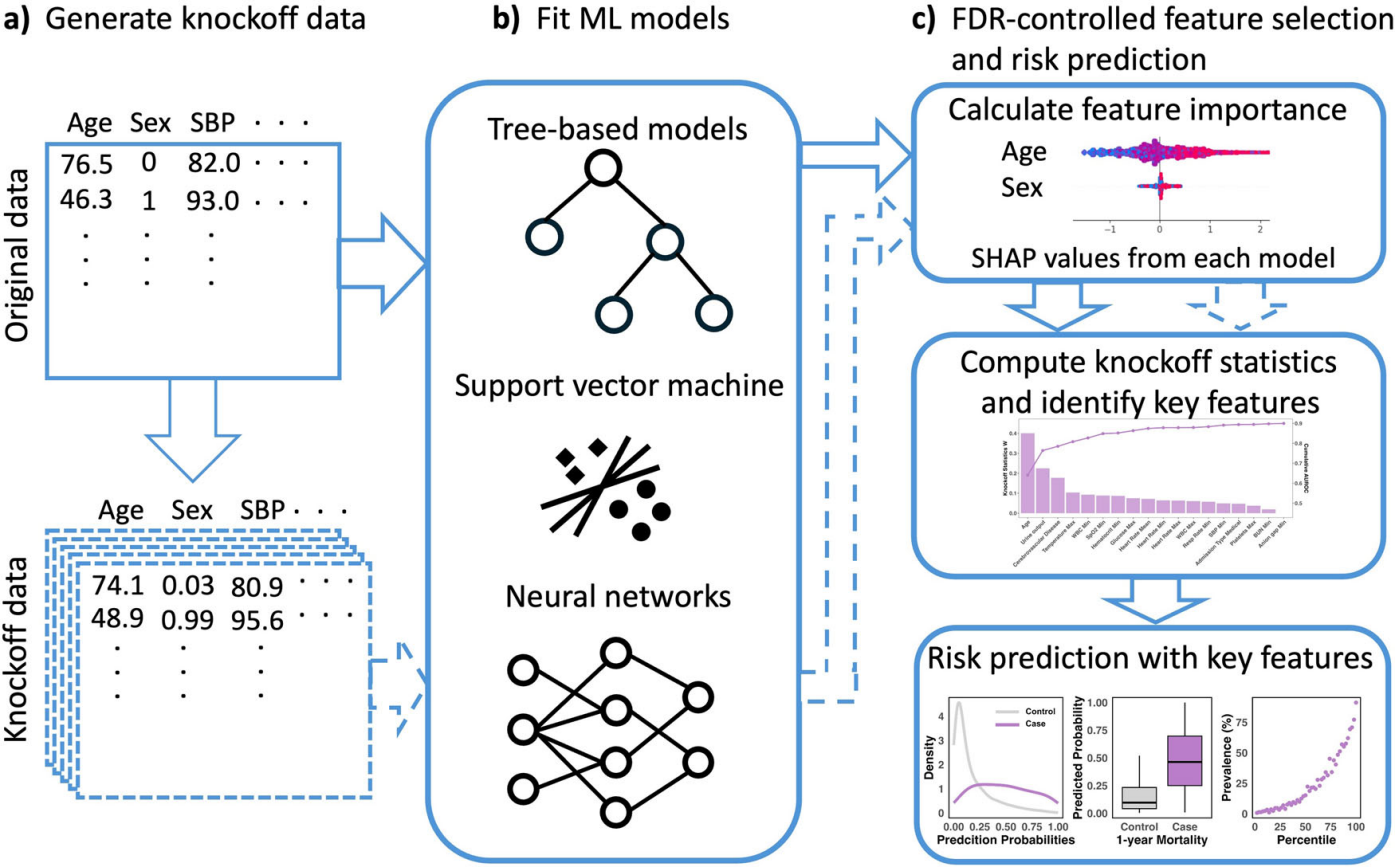
Workflow of Knockoff-ML for feature selection and risk stratification in EHR data. **a** Knockoff-ML generates multiple knockoffs of the original data. **b** Both the original and knockoff features are fed into ML models of choice for model training. Knockoff-ML is flexible to accommodate various types of ML models. **c** Knockoff-ML calculates feature importance for both original and knockoff features using SHAP values and then calculates knockoff statistics to identify key features with FDR control. Knockoff-ML trains ML models with identified risk features for patient risk prediction and stratification. The figures were created with Microsoft PowerPoint (**a**, **b**) and the Python library shap and R library ggplot2 (**c**) and assembled using Microsoft PowerPoint.

We present the workflow of Knockoff-ML in Fig. 1. Knockoff-ML combines the knockoff framework with ML models for controlled variable selection and risk prediction in EHR data. The idea of the knockoff framework is to construct synthetic knockoff features of the original features that are independent of the outcome conditional on the original features. These knockoff features preserve the distribution and correlation structure of the original features so that they can effectively serve as negative controls to identify truly important features. We then use the identified features as input to the ML models for risk prediction. We present the details of the Knockoff-ML workflow in the Methods section.

### Knockoff-ML enables machine learning models to identify risk features with FDR control

**Fig. 2:**
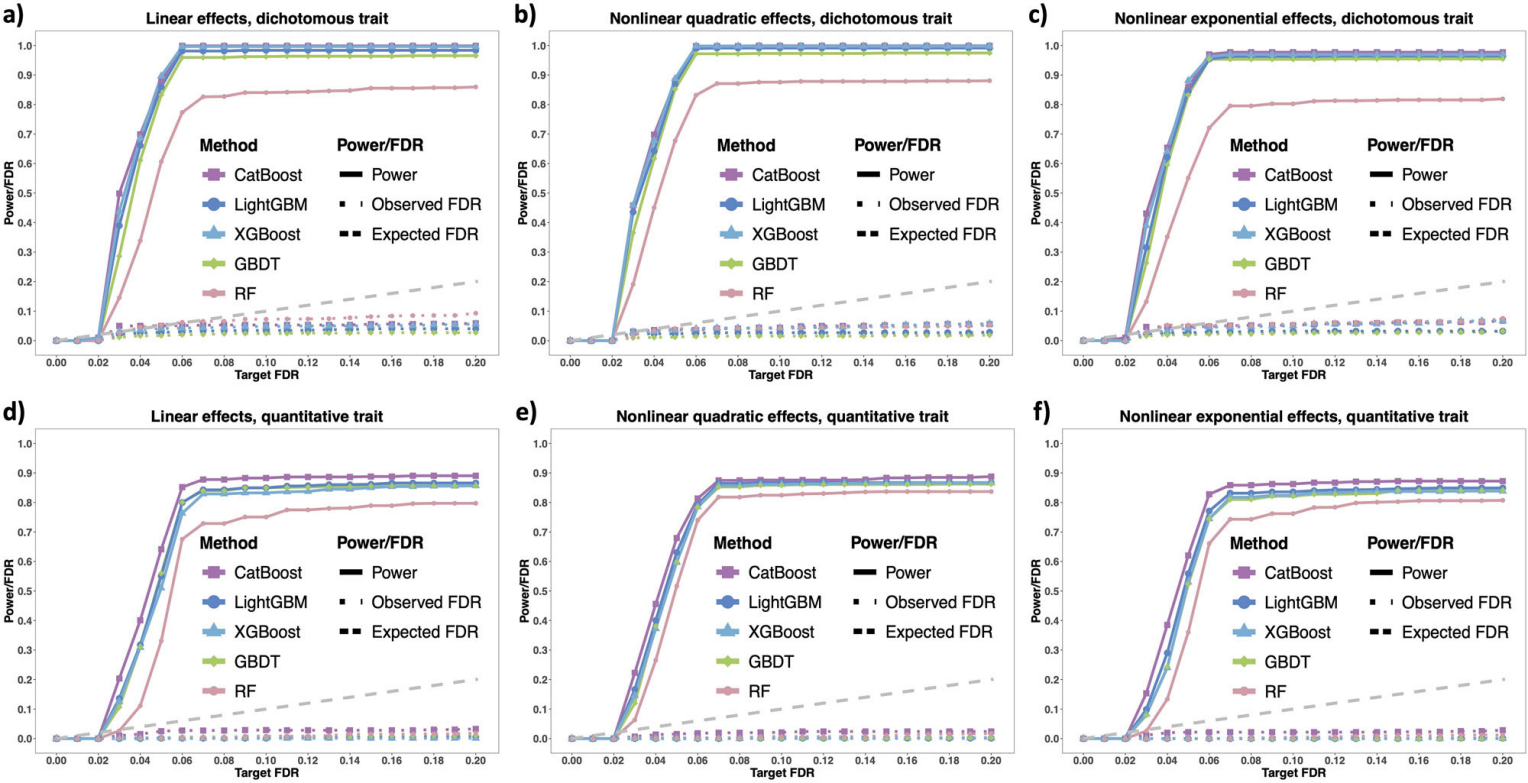
Knockoff-ML’s power and FDR in simulation studies. The six panels show the power and FDR for different traits (**a**–**c** dichotomous traits; and **d**–**f** quantitative traits) and different types of effects (**a**, **d** linear effects; **b**, **e** nonlinear quadratic effects; and **c**, **f** nonlinear exponential effects), with target FDR ranging from 0.01 to 0.20. The solid lines indicate Knockoff-ML's power, and the dotted lines indicate Knockoff-ML's FDR. The different colors and point types indicate different ML models in Knockoff-ML. The gray dashed line indicates the expected FDR. CatBoost: categorical boosting; LightGBM: light gradient boosting machine; XGBoost: eXtreme gradient boosting; GBDT: gradient boosting decision tree; RF: random forest. The figures were created with the R library ggplot2 and assembled using Microsoft PowerPoint.

We performed power and FDR simulations to evaluate Knockoff-ML’s ability to accurately identify risk features for ICU mortality. In each replicate, we generated five knockoff features for each original feature and trained five ML models, including CatBoost, light gradient boosting machine (LightGBM), XGBoost, gradient boosting decision tree (GBDT), and RF, with the original and knockoff features, respectively. We evaluated the power and FDR of Knockoff-ML for both dichotomous and quantitative traits under linear and nonlinear scenarios. Simulation details are provided in the Methods section. We define power as the proportion of identified risk features among all risk features and FDR as the proportion of non-risk features among all identified features. Typically, a method with high power has a high chance of identifying a risk feature (e.g., a method with 80% power can roughly identify four out of five risk features in the data), whereas a method with low FDR has a high confidence for an identified feature to be a risk feature (e.g., roughly only one out of five identified features is not a risk feature for a method with 20% FDR). In each scenario, we simulated 100 replicates and reported the average power and FDR at different target FDR levels ranging from 0.01 to 0.20. As shown in Fig. 2, Knockoff-ML enables the ML models to achieve high power to identify risk features while controlling the FDR at target levels in all scenarios.

We compared the FDR and power of Knockoff-ML with several conventional feature selection methods, such as stepwise regression, backward elimination, forward selection, and least absolute shrinkage and selection operator (lasso) with two different regularization parameters *λ* (i.e., ${\lambda }_{\min }$ and *λ*_1se_). The details of feature selection criteria for these methods are provided in the Methods section. The power and FDR for Knockoff-ML were calculated with features identified at a target FDR of 0.1 using CatBoost. In each scenario, we simulated 100 replicates and reported the average power and FDR for each method. Knockoff-ML exhibited comparable power and a substantially lower FDR than the conventional methods in most scenarios (Supplementary Fig. 1). In particular, Knockoff-ML exhibited higher power than the conventional methods in scenarios with nonlinear quadratic effects (Supplementary Fig. 1b and e). On the other hand, the conventional methods exhibited an inflated FDR in most scenarios. We note that lasso with *λ*_1se_ (lasso-1se) achieved a lower FDR with slightly reduced power than lasso with ${\lambda }_{\min }$ (lasso-min) due to its larger regularization parameter.

### Knockoff-ML achieves superior prediction accuracy with identified features

We further evaluated the predictive power of Knockoff-ML’s identified features by training ML models using these features and comparing the prediction accuracy with the same models trained using all available features (i.e., full models). To measure prediction accuracy, we used the area under the receiver operating characteristic curve (AUROC) for dichotomous traits and root mean square error (RMSE) and R-squared (*R*^2^) for quantitative traits. We report the average of these metrics across 100 replicates for each scenario. Knockoff-ML demonstrated superior predictive performance across all ML models for both dichotomous and quantitative traits. Specifically, prediction models with features identified by Knockoff-ML consistently achieved comparable predictive performance with the full models. For dichotomous traits, the average AUROC of Knockoff-ML models was nearly identical to that of the full models (Supplementary Table 1). For example, the average AUROC was 0.998 for both the full model and Knockoff-ML in the scenario of linear dichotomous traits with CatBoost. We note that the difference in AUROC between Knockoff-ML and the full models was less than 0.005 across all scenarios. This aligns with the power/FDR simulation results for identifying risk features on dichotomous traits, where Knockoff-ML was able to identify almost all risk features with an average power close to 1. Similarly, for quantitative traits, Knockoff-ML models showed comparable predictive performance to the full models, with only a negligible decline in average *R*^2^ (Supplementary Table 2) and with only a negligible increase in average RMSE (Supplementary Table 3). For example, the average *R*^2^ was 0.890 for the full model and was 0.843 for Knockoff-ML in the scenario of linear quantitative traits with CatBoost, while the average RMSE was 1.053 for the full model and was 1.347 for Knockoff-ML in the same scenario.

We compared the predictive performance of Knockoff-ML with the same conventional feature selection methods from the previous section. Specifically, we trained CatBoost using features identified by Knockoff-ML at a target FDR of 0.1 and features identified by the conventional methods, respectively, and reported the average AUROC, RMSE, and *R*^2^ in test sets across 100 replicates for each scenario. Knockoff-ML and the conventional methods have comparable AUROCs (dichotomous traits) and RMSE/*R*^2^ (quantitative traits) for both linear and nonlinear exponential effects (Supplementary Table 4). For nonlinear quadratic effects, we note that Knockoff-ML exhibited higher AUROCs, higher *R*^2^, and lower RMSE than the conventional methods. For example, the AUROC was 0.996 for Knockoff-ML, compared with 0.835 for stepwise regression, 0.832 for backward elimination, 0.809 for forward selection, 0.799 for lasso-min, and 0.735 for lasso-1se. Similarly, the *R*^2^ and RMSE were 0.848 and 1.647 for Knockoff-ML, respectively, whereas the largest *R*^2^ was 0.488 and the lowest RMSE was 3.330 for the conventional methods.

### Application of Knockoff-ML to MIMIC-IV database

#### Data preprocessing and quality control

We applied Knockoff-ML to the Medical Information Mart for Intensive Care (MIMIC)-IV database^19^ to identify risk features for ICU mortality and predict patient risk. The MIMIC-IV dataset contains 73,181 ICU admissions records. We retained only the first admission record for each patient, resulting in a total of 50,591 records/patients in our analysis. The outcomes of interest for risk prediction and stratification were 7-day mortality (mortality status within 7 days after ICU admission), 30-day mortality (mortality status within 30 days after ICU admission), and 1-year mortality (mortality status within 1 year after ICU admission), respectively.

We implemented quality control procedures to filter and impute variables. We first extracted 155 variables related to demographics, vital signs, laboratory tests, and comorbid conditions from MIMIC-IV. For continuous variables, we winsorized at the 1st and 99th percentiles to mitigate the impact of outliers^20^. We excluded 78 variables with over 30% missing values. For 55 continuous variables with a missing rate less than 30%, multiple imputation by chained equations (MICE)^21^ was employed to impute the missing values. All continuous variables were standardized to have a mean of zero and a standard deviation of one. For the only categorical variable with a missing rate below 30%, we imputed its missing values using the mode. We then converted categorical variables into dummy variables, resulting in a total of 88 variables.

#### Knockoff-ML identified risk features for ICU mortality with FDR control

We trained five ML models, including CatBoost, LightGBM, XGBoost, GBDT, and RF, in the Knockoff-ML framework to identify features that significantly influence the mortality of ICU patients with FDR control at the target level of 0.1. Specifically, Knockoff-ML with CatBoost, LightGBM, XGBoost, GBDT, and RF identified 19, 19, 18, 15, and 17 features, respectively, for 7-day mortality (Supplementary Table 5 and Supplementary Fig. 2a). For 30-day mortality, Knockoff-ML with CatBoost, LightGBM, XGBoost, GBDT, and RF identified 22, 19, 23, 15, and 21 features, respectively (Supplementary Table 6 and Supplementary Fig. 2b). For 1-year mortality, Knockoff-ML with CatBoost, LightGBM, XGBoost, GBDT, and RF identified 21, 20, 22, 18, and 24 features, respectively (Supplementary Table 7 and Supplementary Fig. 2c). To unify the feature selection based on different ML models in Knockoff-ML, we calculate the number of times each feature is identified by the five models and define a feature as a risk feature if it is selected by at least three out of the five models. As shown in Table 1, Knockoff-ML identified 18, 17, and 20 risk features for 7-day mortality, 30-day mortality, and 1-year mortality, respectively.

**Table 1:** Risk features identified by Knockoff-ML for ICU mortality

Variable	7-day	30-day	1-year
Demographics			
Age	0.0125	0.0125	0.0111
Gender	-	-	0.0133
Vital Signs			
Heart rate (Mean)	0.0125	0.0125	0.0111
Heart rate (Min)	0.0125	0.0125	0.0111
Heart rate (Max)	0.0125	0.0125	0.0111
Temperature (Max)	0.0125	0.0125	0.0111
SpO2 (Min)	0.0125	0.0125	0.0111
Respiratory rate (Min)	0.0143	-	-
Systolic blood pressure (Min)	0.0154	-	-
Lab Tests			
Blood urea nitrogen (Min)	0.0125	0.0125	0.0111
Hematocrit (Min)	0.0143	0.0125	0.0133
Platelets (Max)	0.0125	0.0125	0.0111
Glucose (Max)	0.0125	0.0125	0.0111
Glucose (Min)	-	0.0125	0.0111
White blood cell (Min)	0.0125	0.0125	0.0111
White blood cell (Max)	0.0125	-	-
Anion gap (Min)	0.0154	-	-
Hemoglobin (Max)	-	0.0154	0.0133
Potassium (Min)	-	-	0.0471
Urine			
Urine output	0.0125	0.0125	0.0111
Comorbidities			
Cerebrovascular disease	0.0125	0.0125	0.0111
Metastatic solid tumor	-	0.0125	-
Malignant cancer	-	-	0.0111
Mild liver disease	-	-	0.0143
Admission Type			
Medical	0.0154	0.0125	0.0250

Features selected by at least three out of the five models in Knockoff-ML are identified as risk features. For each risk feature, we show the minimum knockoff *q* value of Knockoff-ML with different ML models. The “-” symbol indicates that a feature is not identified as a risk feature for an outcome. “Max”, “Mean”, and “Min” indicate the maximum, mean, and minimum values of a risk feature, respectively, within 24 hours after ICU admission.

Our results revealed that the identified risk features have different impacts on short-term and long-term mortality. Age was one of the most significant risk factors for 7-day, 30-day, and 1-year mortality in both our analysis and previous studies^22–24^. Gender was identified as a risk factor for 1-year mortality, with females having a significantly higher 1-year mortality than males, which is in line with a previous meta-analysis study of over 25,000 patients in Canadian and French ICUs^25^. Vital signs are also critical risk factors for ICU mortality. Heart rate^26–28^, body temperature^29^, and SpO2^30,31^ were found to influence both short-term and long-term mortality, which aligns with our findings that all of them were risk features for 7-day, 30-day, and 1-year mortality. We also identified respiratory rate and systolic blood pressure as risk factors for 7-day mortality, which is consistent with previous findings that abnormal respiratory rate^32,33^ and episodes of hypotension during ICU stays^34^ were primarily critical predictors of short-term mortality. Regarding laboratory tests, elevated blood urea nitrogen^35,36^, abnormal levels of hematocrit^37,38^, elevated platelet count^39,40^, both high glucose levels and high glucose variability^41,42^, and a higher white blood cell count^43,44^ were associated with increased short-term or long-term mortality, supporting our findings that all of these features were risk factors for 7-day, 30-day, and 1-year mortality. Early hemoglobin status was found to specifically predict long-term mortality in sepsis patients^45^, aligning with our finding that hemoglobin was only associated with 1-year mortality. Urine output, a common risk factor for in-hospital mortality^46^ and ICU complications such as acute kidney injury^47^, significantly influences both short- and long-term mortality in our analysis. Moreover, we identified four comorbidities that were significantly associated with ICU mortality, three of which were only associated with mid- and long-term mortality. This is consistent with previous studies that demonstrated comorbidities with a more pronounced impact on long-term mortality^48,49^. Specifically, we found that cerebrovascular disease was significantly associated with 7-day, 30-day, and 1-year mortality, while metastatic solid tumor (a malignant tumor) was associated with 30-day mortality and malignant cancer and mild liver disease were associated with 1-year mortality. These findings are consistent with previous studies that cancer was associated with a greater one-year risk of death than non-cancer patients^50^, and malignant tumor was a significant cause of death in post-ICU discharge within 2.6 months^51^. The type of ICU admission was also a risk factor for all mortality outcomes in our analysis, where medical ICUs had significantly higher mortality rates than scheduled surgical and unscheduled surgical ICU admissions. This is consistent with previous findings on medical admissions with observed higher mortality rates than surgical admissions^52^. We showed the ranking of risk features by knockoff statistics for each outcome across different models in Supplementary Fig. 3 and the differences in these risk features between censored patients and patients who experienced the mortality events in Supplementary Tables 8–10.

#### Knockoff-ML accurately predicted ICU mortality with identified risk features

To demonstrate the predictive power of features identified by Knockoff-ML, we re-trained the five ML models, i.e., CatBoost, LightGBM, XGBoost, GBDT, and RF, using only features identified by Knockoff-ML for each mortality outcome and compared the prediction accuracy with models that we trained using all available 88 features (i.e., full models). We used 70% of the dataset as the training set (N = 35,644) and 30% as the test set (N = 15,277) for each outcome, respectively. The proportion of cases (i.e., patients who experienced mortality events) and controls (i.e., censored patients) in the training set was kept equal to that in the test set. The detailed demographics of cases and controls in both the training and test sets for each outcome are summarized in Supplementary Tables 11–13.

**Fig. 3:**
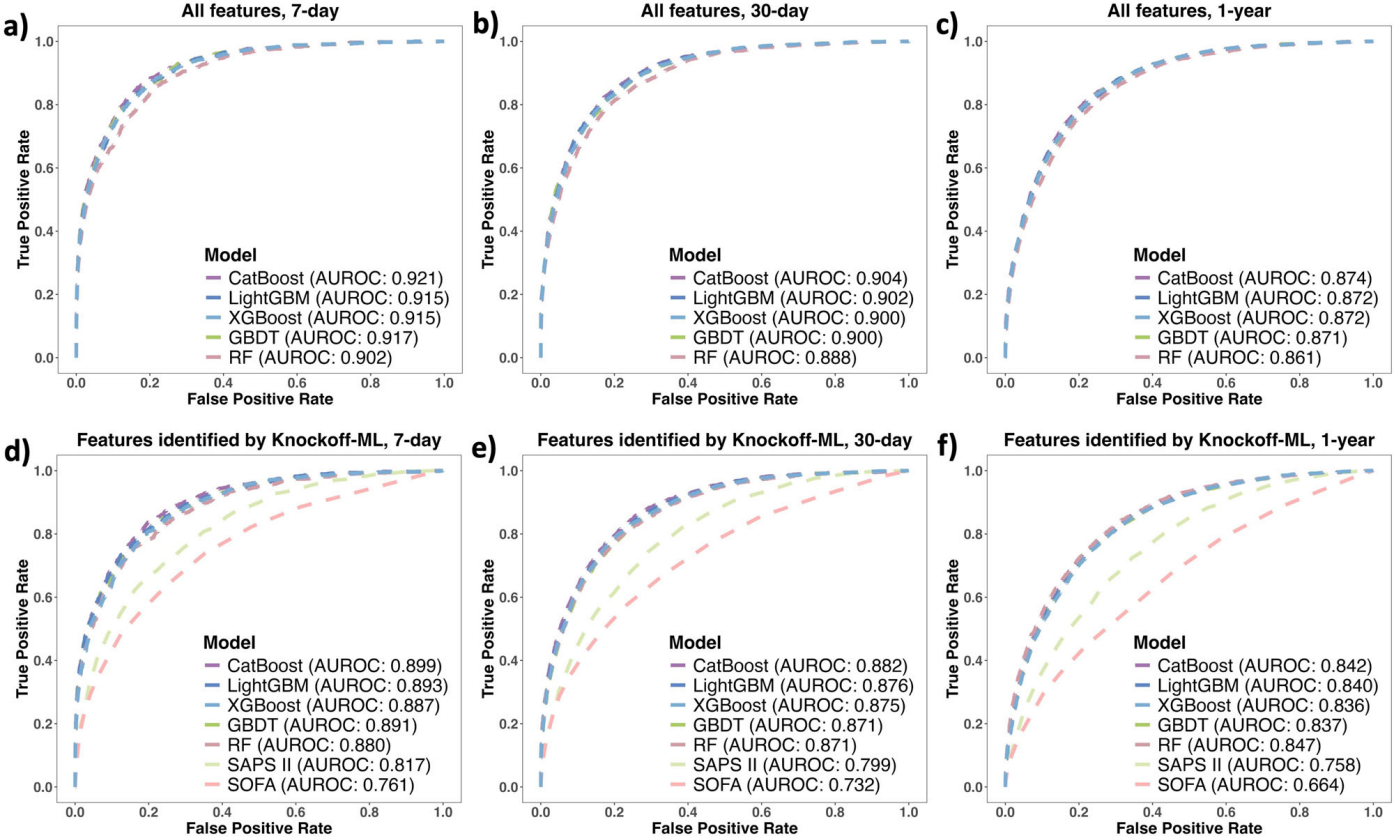
Area under receiver operating characteristic curve (AUROC) for models with all features and features identified by Knockoff-ML. Outcomes of each panel: **a**, **d** 7-day mortality; **b**, **e** 30-day mortality; **c**, **f** 1-year mortality. Features used in models of each panel: **a**–**c** all features; **d**–**f** features identified by Knockoff-ML. All AUROCs are derived from the test set. CatBoost: categorical boosting; LightGBM: light gradient boosting machine; XGBoost: eXtreme gradient boosting; GBDT: gradient boosting decision tree; RF: random forest; SOFA: sequential organ failure assessment; SAPS II: simplified acute physiology score II. The figures were created with the R library ggplot2 and assembled using Microsoft PowerPoint.

Knockoff-ML consistently achieved comparable performance with the full models across all ML models for each outcome with only a negligible decline in AUROC (Fig. 3 and Table 2). For example, using CatBoost for 7-day mortality, the AUROC was 0.92 for the full model and was 0.90 for Knockoff-ML. We implemented the DeLong test^53^ for each pair of prediction models to evaluate the significance of differences in their AUROCs (Supplementary Fig. 4). We note that CatBoost exhibited significantly higher AUROC than the other models in most scenarios. In addition to the full models, we also benchmarked Knockoff-ML against conventional scoring systems, i.e., SOFA^7^ and SAPS II^5^. Compared to these established risk assessment tools, Knockoff-ML consistently demonstrated superior predictive performance for all mortality outcomes (Fig. 3). Furthermore, we compared the prediction accuracy with models that we trained using Knockoff-ML’s lowest-ranked features selected in ascending order of feature importance scores. The number of lowest-ranked features used in each model was set equal to the number of identified features in each model for the corresponding outcome. We note that the AUROC for these models was substantially lower, ranging from 0.58 to 0.76 (Supplementary Fig. 5), than the AUROC for models that we trained using risk features identified by Knockoff-ML. These results demonstrated that Knockoff-ML can identify truly important features that are significant risk factors for ICU mortality, and these risk features have substantially higher predictive power for ICU mortality than the rest of the features in the data.

**Table 2:** Mortality prediction using all and Knockoff-ML-identified features

Outcome	Model	All features			
		AUROC	Sensitivity	Specificity	F1 score
7-day Mortality	CatBoost	0.921 (0.913–0.928)	0.963 (0.960–0.966)	0.617 (0.586–0.648)	0.569 (0.545–0.593)
	LightGBM	0.915 (0.907–0.923)	0.960 (0.957–0.963)	0.613 (0.580–0.643)	0.546 (0.518–0.573)
	XGBoost	0.915 (0.907–0.923)	0.964 (0.961–0.967)	0.548 (0.518–0.575)	0.551 (0.526–0.575)
	GBDT	0.917 (0.908–0.924)	0.960 (0.957–0.964)	0.630 (0.599–0.661)	0.555 (0.526–0.579)
	RF	0.902 (0.893–0.911)	0.962 (0.958–0.965)	0.545 (0.517–0.575)	0.531 (0.506–0.555)
30-day Mortality	CatBoost	0.904 (0.897–0.911)	0.945 (0.942–0.949)	0.564 (0.544–0.584)	0.604 (0.588–0.622)
	LightGBM	0.902 (0.896–0.909)	0.951 (0.947–0.955)	0.536 (0.517–0.555)	0.604 (0.587–0.620)
	XGBoost	0.900 (0.893–0.908)	0.945 (0.941–0.949)	0.546 (0.528–0.568)	0.593 (0.576–0.609)
	GBDT	0.900 (0.893–0.906)	0.946 (0.943–0.950)	0.541 (0.521–0.560)	0.594 (0.576–0.611)
	RF	0.888 (0.880–0.895)	0.941 (0.937–0.945)	0.526 (0.507–0.546)	0.570 (0.552–0.588)
1-year Mortality	CatBoost	0.874 (0.868–0.880)	0.908 (0.902–0.913)	0.598 (0.583–0.612)	0.661 (0.650–0.673)
	LightGBM	0.872 (0.865–0.878)	0.902 (0.896–0.908)	0.595 (0.581–0.609)	0.652 (0.640–0.665)
	XGBoost	0.872 (0.866–0.878)	0.910 (0.904–0.915)	0.579 (0.566–0.593)	0.654 (0.643–0.665)
	GBDT	0.871 (0.864–0.876)	0.917 (0.911–0.922)	0.564 (0.551–0.577)	0.653 (0.642–0.665)
	RF	0.861 (0.855–0.867)	0.908 (0.902–0.913)	0.563 (0.550–0.576)	0.642 (0.631–0.653)

Outcome	Model	Features identified by Knockoff-ML			
		AUROC	Sensitivity	Specificity	F1 score
7-day Mortality	CatBoost	0.899 (0.890–0.908)	0.959 (0.956–0.962)	0.566 (0.536–0.598)	0.521 (0.495–0.547)
	LightGBM	0.893 (0.883–0.902)	0.959 (0.956–0.962)	0.581 (0.549–0.611)	0.525 (0.498–0.550)
	XGBoost	0.887 (0.877–0.897)	0.961 (0.957–0.964)	0.488 (0.460–0.515)	0.500 (0.475–0.524)
	GBDT	0.891 (0.881–0.900)	0.959 (0.955–0.962)	0.572 (0.543–0.604)	0.521 (0.496–0.549)
	RF	0.880 (0.870–0.890)	0.954 (0.951–0.958)	0.594 (0.558–0.629)	0.491 (0.463–0.518)
30-day Mortality	CatBoost	0.882 (0.875–0.890)	0.939 (0.935–0.943)	0.514 (0.494–0.531)	0.558 (0.540–0.574)
	LightGBM	0.876 (0.868–0.884)	0.940 (0.936–0.944)	0.483 (0.464–0.501)	0.545 (0.528–0.563)
	XGBoost	0.875 (0.867–0.882)	0.940 (0.936–0.944)	0.489 (0.470–0.508)	0.548 (0.530–0.566)
	GBDT	0.871 (0.863–0.879)	0.940 (0.936–0.945)	0.477 (0.459–0.497)	0.542 (0.524–0.559)
	RF	0.871 (0.863–0.879)	0.938 (0.933–0.942)	0.493 (0.474–0.512)	0.543 (0.525–0.560)
1-year Mortality	CatBoost	0.842 (0.835–0.848)	0.892 (0.886–0.898)	0.556 (0.541–0.570)	0.619 (0.606–0.630)
	LightGBM	0.840 (0.833–0.846)	0.896 (0.890–0.902)	0.538 (0.524–0.552)	0.614 (0.602–0.626)
	XGBoost	0.836 (0.828–0.843)	0.902 (0.896–0.907)	0.513 (0.501–0.527)	0.607 (0.596–0.619)
	GBDT	0.837 (0.830–0.844)	0.895 (0.889–0.901)	0.539 (0.525–0.553)	0.614 (0.602–0.624)
	RF	0.847 (0.840–0.853)	0.899 (0.893–0.905)	0.540 (0.526–0.555)	0.619 (0.608–0.630)

Sensitivity the proportion of true positives that were predicted as positive, Specificity the proportion of true negatives that were predicted as negative, F1-score the harmonic mean of recall and precision, where recall is sensitivity and precision is the proportion of correctly classified positive cases out of all predicted positives.

*AUROC* area under receiver operating characteristic curve, *CatBoost* categorical boosting, *LightGBM* light gradient boosting machine, *XGBoost* eXtreme gradient boosting, *GBDT* gradient boosting decision tree, *RF* random forest.

We further compared the predictive performance of Knockoff-ML with the conventional feature selection methods (i.e., stepwise regression, backward elimination, forward selection, lasso-min, and lasso-1se). For 7-day mortality, stepwise regression, backward elimination, forward selection, lasso-min, and lasso-1se identified 59, 59, 58, 79, and 58 features, respectively (Supplementary Fig. 2a). For 30-day mortality, stepwise regression, backward elimination, forward selection, lasso-min, and lasso-1se identified 61, 61, 63, 81, and 62 features, respectively (Supplementary Fig. 2b). For 1-year mortality, stepwise regression, backward elimination, forward selection, lasso-min, and lasso-1se identified 66, 66, 63, 84, and 55 features, respectively (Supplementary Fig. 2c). We then trained the five ML models using these identified features from each method for each outcome, and compared their AUROC with that of the full models and Knockoff-ML (Supplementary Figs. 6–10). The ML models trained with features identified by the conventional methods exhibited AUROC that were comparable to the full models and higher than Knockoff-ML (Panels a, b, and c in Supplementary Figs. 6–10). One plausible explanation is that the number of features identified by the conventional methods was excessively large compared to Knockoff-ML (Supplementary Fig. 2). For a fairer comparison, we re-trained the ML models with the highest-ranked features selected in descending order of the absolute values of coefficient estimates $| \hat{\beta }|$ for the conventional methods. The number of highest-ranked features used in each model was set equal to the number of features identified by Knockoff-ML with corresponding ML models for each outcome. In this case, Knockoff-ML demonstrated higher AUROCs across most models for both 7-day mortality and 30-day mortality and comparable AUROCs for 1-year mortality compared to most conventional methods (Panels d, e, and f in Supplementary Figs. 6–10).

**Fig. 4:**
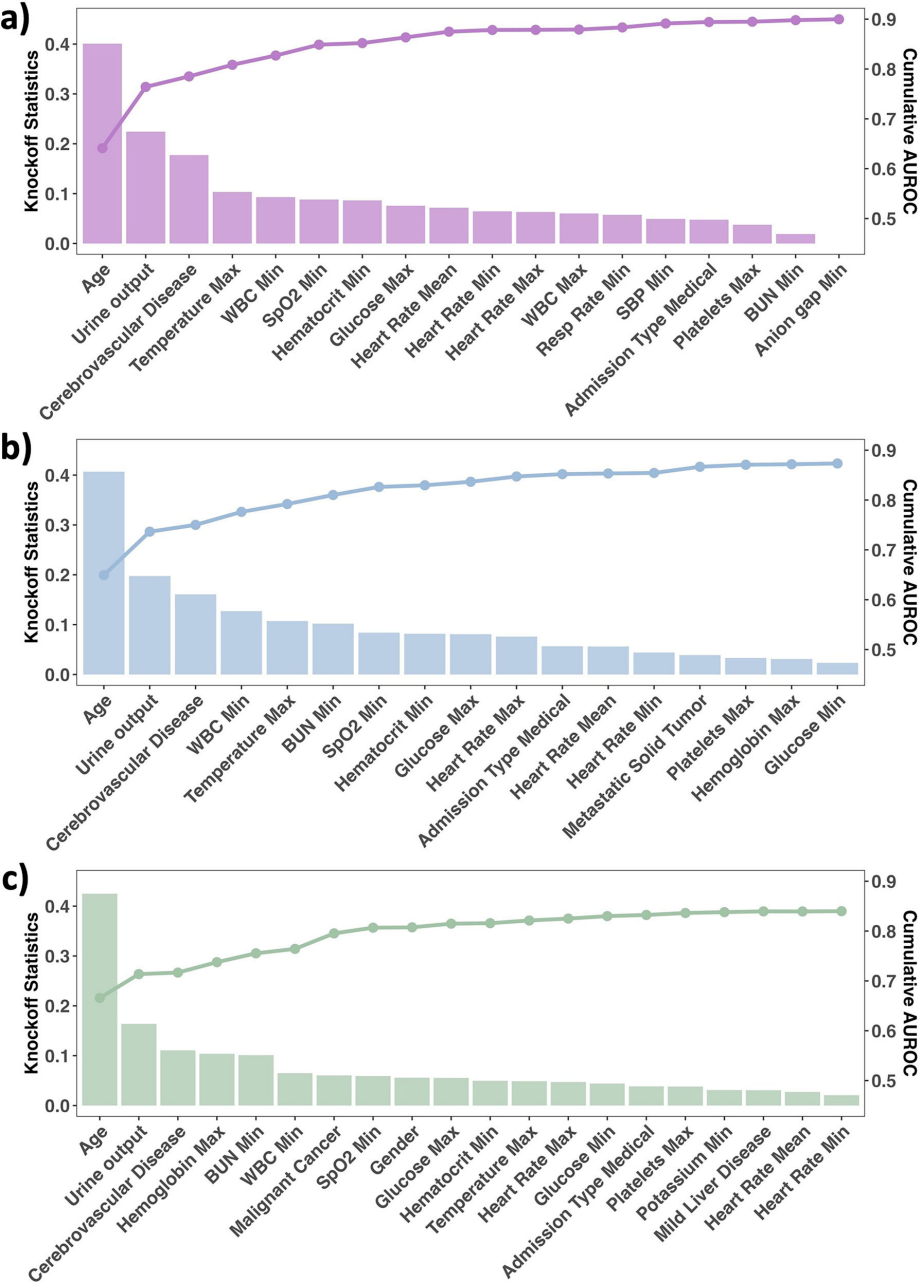
Knockoff statistics for risk features and cumulative AUROC. The bars show knockoff statistics and the lines show the cumulative AUROC. The features are ranked in descending order based on their respective knockoff statistics. Outcomes of each panel: **a** 7-day mortality; **b** 30-day mortality; **c** 1-year mortality. “Max”, “Mean”, and “Min” indicate the maximum, mean, and minimum values of a risk feature, respectively, within 24 h after ICU admission. WBC white blood cell, SBP systolic blood pressure, BUN blood urea nitrogen, Resp Rate: respiratory rate. The figures were created with the R library ggplot2 and assembled using Microsoft PowerPoint.

We trained the final prediction models using CatBoost with the 18, 17, and 20 risk features unified across different ML models in Knockoff-ML for each mortality outcome, respectively. To evaluate the contribution of each risk feature to the model’s predictive power, we sequentially added the risk features to CatBoost in descending order of knockoff statistics and calculated the AUROC each time we added a feature. As shown in Fig. 4, the AUROC consistently increases with each risk feature being added, while the rate of increase in the AUROC decreases as the knockoff statistics decrease. We also examined the estimated distribution of predicted probabilities from the final prediction models based on risk features identified by Knockoff-ML for cases (i.e., patients who experienced mortality events) and controls (i.e., censored patients). As shown in Supplementary Fig. 11, the predicted probabilities for cases were significantly higher than those for controls, and the proportion of cases consistently increased as the predicted probability increased. These results indicate that Knockoff-ML identifies risk features that are truly important risk factors for ICU mortality, and produces knockoff statistics that are reflective of the importance of risk features.

#### Knockoff-ML improved clinical risk stratification relative to conventional methods

**Fig. 5:**
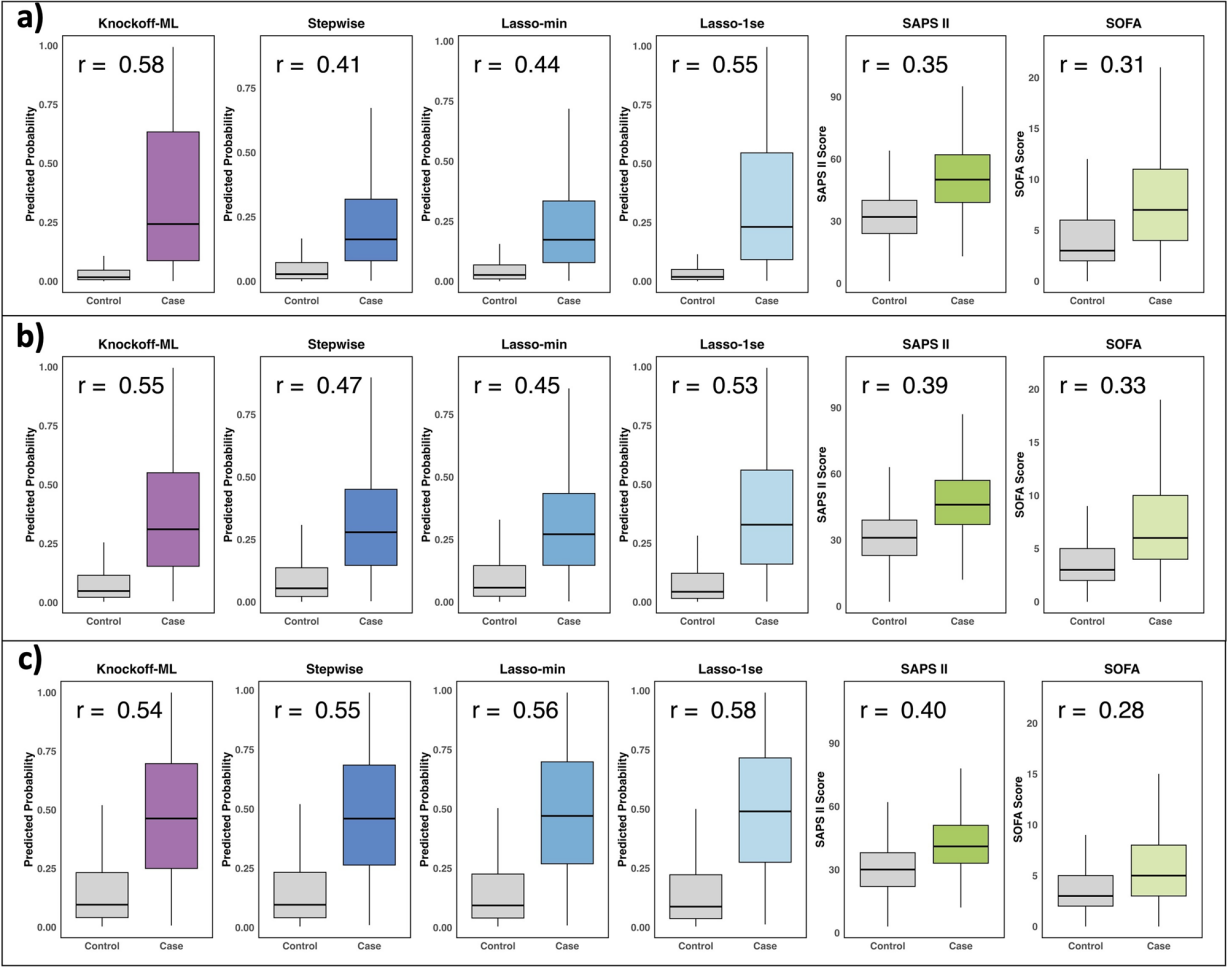
Knockoff-ML improved risk prediction relative to conventional methods. Boxplots of predicted probabilities, SAPS II scores, and SOFA scores. All predicted probabilities were obtained from CatBoost models trained with features identified by Knockoff-ML, stepwise regression, lasso-min, and lasso-1se in the test set. The number of the highest-ranked features based on the absolute values of coefficient estimates $| \hat{\beta }|$ from stepwise regression, lasso-min, and lasso-1se used in CatBoost models was equal to the number of risk features identified by Knockoff-ML for the corresponding outcome. The center line, lower and upper bounds of the box indicate the median, the 25th percentile (*Q*_1_), and the 75th percentile (*Q*_3_) of the data. The whiskers extend upward (downward) to the highest (lowest) observed data point that falls within 1.5 times the interquartile range (*Q*_3_ − *Q*_1_) above the *Q*_3_ and below the *Q*_1_. *r* is the point-biserial correlation coefficient. Outcomes of each panel: **a** 7-day mortality; **b** 30-day mortality; **c** 1-year mortality. CatBoost: categorical boosting; Lasso-min: lasso with the regularization parameter ${\lambda }_{\min }$ that gives the minimum mean cross-validation error; Lasso-1se: lasso with the regularization parameter *λ*_1se_ that gives the most regularized model such that the cross-validation error is within one standard error of the minimum. The figures were created with the R library ggplot2 and assembled using Microsoft PowerPoint.

The predicted probabilities from the final prediction models based on risk features identified by Knockoff-ML demonstrate a strong correlation with mortality outcomes (Fig. 5). To compare with conventional methods, we trained CatBoost models with the highest-ranked features selected in descending order of the absolute values of coefficient estimates $| \hat{\beta }|$ for stepwise regression, lasso-min, and lasso-1se, respectively, with the number of features being set equal to the number of risk features in Knockoff-ML’s final prediction models. For 7-day mortality, the predicted probabilities from Knockoff-ML achieved a point-biserial correlation^54^ of 0.58 with mortality outcomes, which is higher than stepwise regression (0.41), lasso-min (0.44), lasso-1se (0.55), SAPS II (0.35), and SOFA (0.31). We observed similar results for 30-day mortality. For 1-year mortality, Knockoff-ML exhibited a point-biserial correlation that was comparable to stepwise regression and lasso and was higher than SAPS II and SOFA.

We further evaluated the clinical utility of our final prediction models using decision curve analysis (DCA)^55^. DCA calculates a “net benefit” to assess the clinical value for prediction models. The net benefit is defined as $\frac{\#\{\,{{\text{true}}\, {\text{positives}}}\,\}}{n}-\frac{\#\{\,{{\text{false}}\, {\text{positives}}}\,\}}{n}\times \frac{{p}_{t}}{1-{p}_{t}}$, where *n* is the number of patients and *p*_*t*_ is a threshold probability. For a prediction model that yields predicted probability $\hat{p}$, we classify patients with $\hat{p}\ge {p}_{t}$ as cases at a given *p*_*t*_ and then calculate the net benefit. Unlike AUROC, the net benefit incorporates the clinical consequences of decisions made based on the model’s predictions, with higher net benefits indicating better clinical utility.

**Fig. 6:**
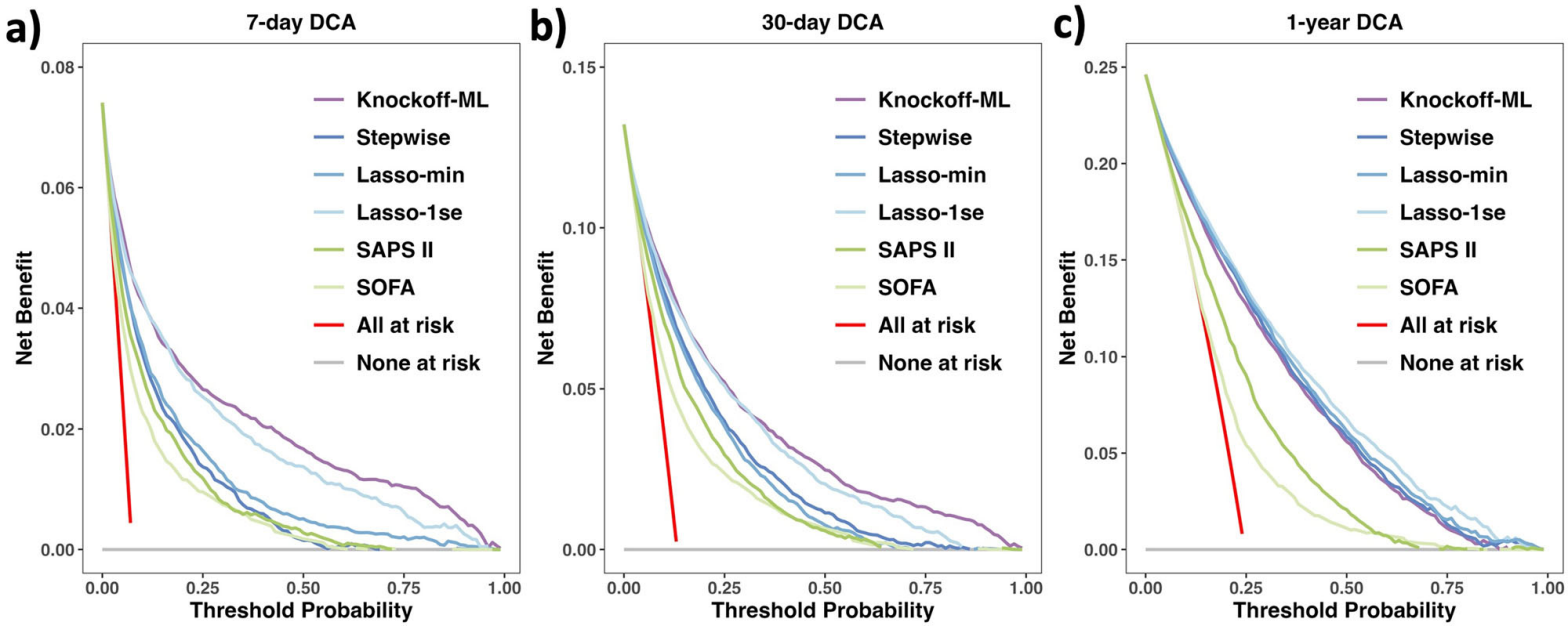
Knockoff-ML improved net benefits over conventional methods. The *X*-axis indicates the threshold probability and the *Y*-axis indicates the net benefit. For Knockoff-ML, stepwise regression, lasso-min, and lasso-1se, the predicted probabilities used in DCA were obtained from CatBoost models trained with identified features in the test set, respectively. The CatBoost models were trained using the highest-ranked features selected in descending order of the absolute values of coefficient estimates $| \hat{\beta }|$ for stepwise regression, lasso-min, and lasso-1se, with the number of features set equal to the number of risk features identified by Knockoff-ML for corresponding outcomes. For SAPS II and SOFA, the predicted probabilities were obtained from logistic regressions of mortality outcomes on SAPS II and SOFA scores. “All at risk” refers to treating all patients as cases and “None at risk” refers to treating no patients as cases. Outcomes of each panel: **a** 7-day mortality; **b** 30-day mortality; **c** 1-year mortality. CatBoost: categorical boosting; Lasso-min: lasso with the regularization parameter ${\lambda }_{\min }$ that gives the minimum mean cross-validation error; Lasso-1se: lasso with the regularization parameter *λ*_1se_ that gives the most regularized model such that the cross-validation error is within one standard error of the minimum. The figures were created with the R library ggplot2 and assembled using Microsoft PowerPoint.

We compared the net benefits of Knockoff-ML against the conventional feature selection methods (i.e., stepwise regression, lasso-min, and lasso-1se), the conventional scoring systems (i.e., SAPA II and SOFA), and the default strategies of assuming that all or no patients are positive. For Knockoff-ML, stepwise regression, lasso-min, and lasso-1se, we used the predicted probabilities from the final prediction models, respectively. For SAPS II and SOFA, we used the predicted probabilities from logistic regressions of mortality outcomes on SAPS II or SOFA scores. The net benefit of each model across a range of threshold probabilities is presented in a decision curve (Fig. 6). The DCA results demonstrated that the net benefits of Knockoff-ML consistently exceeded those of stepwise regression, lasso-min, lasso-1se, SAPS II, and SOFA across all evaluated threshold ranges for 7-day mortality and 30-day mortality. For 1-year mortality, Knockoff-ML exhibited a net benefit that was comparable to stepwise regression and lasso-min, lower than lasso-1se, and higher than SAPS II and SOFA. Knockoff-ML also had much higher net benefits compared to the default strategies of either treating all or no patients as positive across all outcomes.

## Discussion

In this study, we introduced Knockoff-ML, a knockoff-augmented machine learning framework designed for controlled variable selection and risk stratification in EHR data. Knockoff-ML integrates ML models with the knockoff framework to perform variable selection under rigorous FDR control and subsequently leverages these identified variables to construct predictive models for patient risk stratification. Through comprehensive simulations in both linear and nonlinear scenarios, we demonstrated that Knockoff-ML achieved high power to identify risk features while maintaining controlled FDR, whereas conventional methods such as stepwise regression and lasso exhibited an inflated FDR in most scenarios. In a real-data application to the MIMIC-IV database, Knockoff-ML effectively identified clinically significant risk features and achieved accurate predictions of ICU mortality with identified risk features, significantly outperforming conventional ICU scoring systems such as SAPS II and SOFA.

Built upon the recently developed knockoff framework, Knockoff-ML performs variable selection at target FDR levels and thus offers an objective criterion for identifying risk features in EHR data. The knockoff framework is highly flexible and can leverage a wide range of feature importance from statistical and machine learning methods for controlled variable selection in both linear and nonlinear scenarios. In our study, Knockoff-ML integrates the knockoff framework with ML models that can effectively capture the complex nonlinear relationships between patient features and outcomes. As a result, Knockoff-ML brings together the advantages of both the knockoff framework and ML models so that it can identify risk features in nonlinear associations with patient outcomes while maintaining rigorous FDR control. In comparison, conventional feature selection methods, such as stepwise regression and lasso, often assume linearity and no multicollinearity. However, such assumptions may be violated in EHR data due to potential nonlinear associations between features and outcomes and correlations among vital signs (Supplementary Fig. 12). This can lead to biased coefficient estimates, which in turn result in decreased power and/or an inflated FDR for the conventional feature selection methods (Supplementary Fig. 1). The choice of the regularization parameter *λ* plays an important role in the performance of lasso. A larger *λ* (e.g., *λ*_1se_) reduces the number of selected variables and lowers the FDR for lasso in most scenarios. We note that the FDR for lasso-1se is marginally lower than lasso-min for linear effects with dichotomous traits because ${\lambda }_{\min }$ and *λ*_1se_ are very close to each other in this scenario and thus lead to a similar average number of selected variables (Supplementary Table 14 and Supplementary Fig. 13).

Controlled feature selection via Knockoff-ML heavily relies on robust estimation of feature importance. To ensure a reliable feature selection process, Knockoff-ML uses SHAP, a consistent and interpretable method designed for evaluating feature contributions to model predictions in ML models. Unlike other interpretability tools such as local interpretable model-agnostic explanations^56^, permutation importance^57^, or method-specific approaches like deep learning important features^58^, SHAP stands out due to its key advantages: a strong theoretical foundation from game theory guaranteeing fair and consistent attribution^14^, and its model-agnostic nature applicable across diverse ML models^14,15,59,60^. However, the computation of SHAP values can be computationally expensive, particularly when dealing with large datasets or highly complex ML models. We optimized the process by employing the shrinkage leveraging estimator (SLEV)^61^, a subsampling method that significantly reduced computational burden. For scenarios with smaller datasets or less complex models, direct calculation of SHAP values using the entire sample remains a feasible approach.

We applied Knockoff-ML to identify risk features and predict mortality risk in the ICU using the MIMIC-IV dataset. In our analysis, Knockoff-ML identified 18, 17, and 20 risk features significantly associated with 7-day, 30-day, and 1-year mortality, respectively. Age was identified as the most significant predictor of mortality, affecting both short-term and long-term mortality. Additionally, vital signs and laboratory tests were found to have a stronger impact on short-term mortality, while comorbidities played a more critical role in determining longer-term mortality outcomes. Prediction models trained using risk features identified by Knockoff-ML achieved comparable predictive performance with the models using all available features. Although models trained with features identified by the conventional methods initially achieved a higher AUROC than Knockoff-ML due to the much larger number of identified features, Knockoff-ML still demonstrated a higher AUROC when the number of features identified by the conventional methods used in prediction models was set equal to the number of risk features identified by Knockoff-ML. We also compared Knockoff-ML with widely used ICU scoring systems, including SOFA^7^ and SAPS II^5^. Knockoff-ML with identified risk features achieved a significantly higher AUROC. Moreover, DCA also showed the strong clinical utility of Knockoff-ML, as evidenced by its higher net benefit than SOFA and SAPS II. It is worth mentioning that, unlike conventional scoring systems with predefined, clinically-weighted features designed for specific outcomes, Knockoff-ML dynamically identifies the most significant features and is applicable to various clinical outcomes, which enables a more data-driven and adaptive prediction framework.

We should acknowledge several limitations of our study. First, while Knockoff-ML is designed to be compatible with a wide range of ML and deep learning models, our investigation explored its application with only five specific ML models. Second, the efficient measurement of feature importance in ML models remains an active area of research. Although we improved the computational efficiency of SHAP by SLEV, there is a continuous need to develop accurate and computationally efficient methods for quantifying feature importance, especially in the context of complex ML models and large-scale datasets. Third, our real-data application was performed in a single database on ICU patients. Future studies are necessary to validate our method in additional datasets with different populations and clinical settings. Finally, our current study identified risk features and predicted the overall mortality risk among all patients admitted to the ICU. Subsequent investigations are warranted to identify features associated with mortality risk in ICU patients stratified by specific diseases or those recovering from particular surgical procedures.

In conclusion, we propose a robust and interpretable knockoff-augmented machine learning framework for controlled variable selection and risk prediction in EHR data. Our method effectively identifies critical risk features and accurately predicts patient outcomes, thereby assisting clinicians in making more accurate diagnoses and prognoses and enhancing patient stratification.

## Methods

### Knockoff-ML workflow

In Knockoff-ML, we first construct multiple knockoff features for each feature based on the sequential conditional independent tuples (SCIT)^62,63^ algorithm. The original and knockoff features are then, respectively, fed into the ML models of choice as inputs. Knockoff-ML is flexible to accommodate various types of ML models, such as neural networks, support vector machines, and tree-based models. After model training, we compute the feature importance (FI) by SHAP values, which quantify the influence of a feature on the outcome in ML models. We then calculate a knockoff statistic for each feature based on the FI of both the original and knockoff features and conduct variable selection based on the knockoff statistics to identify risk features for the outcome with FDR control that guarantees most of the identified features are truly risk factors. Lastly, we train ML models using identified risk features for patient risk prediction and stratification. We summarize the steps of Knockoff-ML as follows and present the details of knockoff construction, FI calculation, FDR-controlled feature selection, and ML models for outcome prediction in the following sections.

**Table:** 

**Step 1:** Generate knockoff features.
**Step 2:** Train ML models and compute FIs for both original and knockoff features using SHAP values.
**Step 3:** Compute the knockoff statistics and knockoff *q* values by contrasting the FI of the original features with those of their corresponding knockoff counterparts.
**Step 4:** Apply a knockoff filter to select features with a knockoff statistic larger than an FDR-adaptive threshold, or equivalently, with a knockoff *q* value less than the target FDR level.
**Step 5:** Train ML models using the selected features for risk prediction.

### Knockoff feature generation

A single copy of knockoff features $\tilde{{\boldsymbol{X}}}\in {{\mathbb{R}}}^{n\times p}$ is constructed with the same dimensions as the original features ${\boldsymbol{X}}\in {{\mathbb{R}}}^{n\times p}$, where *p* denotes the number of features and *n* denotes the total number of individuals. Knockoffs are designed to be conditionally independent of the response vector ${\boldsymbol{y}}\in {{\mathbb{R}}}^{n}$. This is done so that truly important features will demonstrate a stronger association with the response when compared to their knockoff counterparts. However, a single knockoff has limited power to identify important features due to the detection threshold $\frac{1}{q}$, i.e., the number of independent signals required for making any discoveries at the target FDR *q*^62^. Specifically, there is no power at the target FDR *q* if there are fewer than $\frac{1}{q}$ discoveries to be made. This is particularly problematic when *q* is low or the signal is sparse. Moreover, the randomness in the generation of a single knockoff leads to instability in the set of selected features. Therefore, to further improve the stability and power of knockoff-based feature selection, we followed the SCIT algorithm^62,63^ to generate multiple knockoff copies. The SCIT algorithm is defined as follows:

#### Algorithm 1

**SCIT algorithm for multiple-knockoff generation**

*j* = 1

**While** *j* ≤ *p* **do**

 Sample ${\tilde{{\boldsymbol{X}}}}_{j}^{1},\ldots ,{\tilde{{\boldsymbol{X}}}}_{j}^{M}$ independently from ${\boldsymbol{L}}({{\boldsymbol{X}}}_{j}| {{\boldsymbol{X}}}_{-j},{\tilde{{\boldsymbol{X}}}}_{1\le k\le j-1}^{1},\ldots ,{\tilde{{\boldsymbol{X}}}}_{1\le k\le j-1}^{M})$

 *j* = *j* + 1

**End**

where − *j* indicates the features ***X***_*j*_ is excluded and *M* is the total number of knockoffs. In line with previous work^64,65^, we adopted a semiparametric model

1 $${{\boldsymbol{X}}}_{j}={\beta }_{0}+\sum _{k\ne j}{\beta }_{k}{{\boldsymbol{X}}}_{k}+\sum _{1\le m\le M}\sum _{k\le j-1}{\gamma }_{k}^{m}{\tilde{{\boldsymbol{X}}}}_{k}^{m}+{\epsilon }_{j}$$

for ${\boldsymbol{L}}({{\boldsymbol{X}}}_{j}| {{\boldsymbol{X}}}_{-j},{\tilde{{\boldsymbol{X}}}}_{1\le k\le j-1}^{1},\ldots ,{\tilde{{\boldsymbol{X}}}}_{1\le k\le j-1}^{M})$, where *ϵ*_*j*_ is a random error term with a mean of zero. We obtain $\hat{{\boldsymbol{\beta }}}$, $\hat{{\boldsymbol{\gamma }}}$, fitted values ${\hat{{\boldsymbol{H}}}}_{j}$, residuals ${\hat{{\boldsymbol{\epsilon }}}}_{j}={{\boldsymbol{H}}}_{j}-{\hat{{\boldsymbol{H}}}}_{j}$, and its *M* permutations ${\hat{{\boldsymbol{\epsilon }}}}_{j}^{* 1},\ldots ,{\hat{{\boldsymbol{\epsilon }}}}_{j}^{* M}$ by minimizing the mean squared loss. We then define the *m*-th knockoff as ${\tilde{{\boldsymbol{H}}}}_{j}^{m}={\hat{{\boldsymbol{H}}}}_{j}+{\hat{{\boldsymbol{\epsilon }}}}_{j}^{* m}$.

### Feature importance in machine learning models

To obtain the FI for both the original and knockoff features, we employed five tree-based ML models, including CatBoost, LightGBM, XGBoost, GBDT, and RF. While these ML models provide inherent feature importance measures, such measures are typically model-specific and lack consistency across different algorithms. To address this limitation, Knockoff-ML uses SHAP^14^, a model-agnostic method that offers a consistent measure of feature importance across different ML models by computing an additive feature importance score^14^ for each variable based on cooperative game theory. Specifically, we denote by *f* the original prediction model and by *g* an explanation model for *f* such that $g({\boldsymbol{z}})={\phi }_{0}+\mathop{\sum }\nolimits_{j = 1}^{p}{\phi }_{j}{z}_{j}$, where ***z*** ∈ {0, 1}^*p*^ with *z*_*j*_ = 1 if the *j*-th variable is present and *z*_*j*_ = 0 if the *j*-th variable is absent. *ϕ*_*ij*_ is the feature attribution value (i.e., the SHAP value) for the *i*-th subject at the *j*-th feature, which can be obtained using the following formula that requires evaluating all possible coalitions of feature values with and without the *j*-th feature:

2 $${\phi }_{ij}=\sum _{{{\boldsymbol{x}}}_{i,S}\subseteq {{\boldsymbol{x}}}_{i,F\setminus \{j\}}}\frac{| {{\boldsymbol{x}}}_{i,S}| !\,(| {{\boldsymbol{x}}}_{i,F}| -| {{\boldsymbol{x}}}_{i,S}| -1)!}{| {{\boldsymbol{x}}}_{i,F}| !}\left[f({{\boldsymbol{x}}}_{i,S\cup \{j\}})-f({{\boldsymbol{x}}}_{i,S})\right],$$

where ***x***_*i*,*F*_ is the vector of all features for the *i*-th sample, ***x***_*i*,*S*_ is a subset of ***x***_*i*,*F*_ not containing the *j*-th feature, and *f*(***x***_*i*,*S*∪{*j*}_) − *f*(***x***_*i*,*S*_) represents the marginal contribution of the *j*-th feature to the coalition. The exact computation of SHAP values is challenging because the number of possible coalitions exponentially increases as more features are included in a prediction model. Lundberg and Lee^14^ proposed Kernel SHAP, which enables regression-based and model-agnostic estimation of SHAP values. Lundberg, Erion, and Lee^15^ then proposed Tree SHAP for tree-based ML models, which offers a fast and model-specific alternative to Kernel SHAP. In practice, we used Tree SHAP for the calculation of SHAP values. FI is obtained by taking the mean absolute SHAP values:

3 $${{\rm{FI}}}_{j}=\frac{1}{n}\mathop{\sum }\limits_{i=1}^{n}\left| {\phi }_{ij}\right| ,$$

where FI_*j*_ is the feature importance score for feature *j*, *ϕ*_*ij*_ is the SHAP value of feature value *x*_*ij*_ for the *i*-th sample and *j*-th feature, and *n* is the total number of samples. Additionally, we performed subsampling to select an informative subset of individuals for the calculation of SHAP values because computing SHAP values for all samples can be computationally expensive, particularly when the sample size is large. Specifically, we adopted the SLEV^61^ to randomly select *k* samples using an importance sampling distribution *π*_*i*_, defined as:

4 $${\pi }_{i}=0.5{\pi }_{i}^{{\rm{Lev}}}+0.5{\pi }_{i}^{{\rm{Unif}}},$$

where ${\pi }_{i}^{{\rm{Lev}}}$ denotes a distribution that reflects the importance of each sample via normalized leverage scores and ${\pi }_{i}^{{\rm{Unif}}}$ denotes the uniform distribution *U*(0, 1). Specifically, ${\pi }_{i}^{{\rm{Lev}}}=\frac{{h}_{ii}}{\sum {h}_{ii}},$ where ${h}_{ii}=\mathop{\sum }\nolimits_{j = 1}^{p}{{\boldsymbol{U}}}_{ij}^{2}$ is the empirical statistical leverage scores; *p* is the total number of features and ***U*** is the leading $\sqrt{p\log p}$^66^ orthogonal singular vectors of (***1***, ***X***) computed via the partial singular value decomposition^67^. In this way, we sample individuals according to an importance sampling distribution *π*_*i*_ that is proportional to the statistical leverage scores of the data matrix ***X***. In practice, we sample $k=\lfloor 10{n}^{1/3}\log n\rfloor$^66^ individuals, where *n* is the total sample size. We performed this subsampling procedure and then computed the SHAP values using selected individuals for the original variables and their corresponding knockoffs.

### Knockoff statistics for feature selection

We denote the FI for the original features by ${{\boldsymbol{T}}}^{0}={({\text{FI}}_{1}^{0},\ldots ,{\text{FI}}_{p}^{0})}^{T}\in {{\mathbb{R}}}^{p}$, and the FI for *M* sets of knockoff features by ${{\boldsymbol{T}}}^{1}={({\text{FI}}_{1}^{1},\ldots ,{\text{FI}}_{p}^{1})}^{T},\ldots ,{{\boldsymbol{T}}}^{M}={({\text{FI}}_{1}^{M},\ldots ,{\text{FI}}_{p}^{M})}^{T}\in {{\mathbb{R}}}^{p}$. Intuitively, original features with higher FI than their knockoffs are more likely to be risk features. For the *j*-th feature, Knockoff-ML calculates a feature statistic defined as:

5 $${W}_{j}=\left({T}_{j}^{0}-\mathop{{\rm{median}}}\limits_{1\le m\le M}{T}_{j}^{m}\right){I}_{{T}_{j}^{0}\ge \mathop{\max }\limits_{1\le m\le M}{T}_{j}^{m}},$$

where *W*_*j*_ is the knockoff statistic *W* for the *j*-th variable, ${T}_{j}^{0}$ and ${T}_{j}^{m}$ are the importance scores computed for the original and the *m*-th knockoff features, respectively, and *I* is the indicator function with ${I}_{{T}_{j}^{0}\ge \mathop{\max }\limits_{1\le m\le M}{T}_{j}^{m}}=1$ if ${T}_{j}^{0}\ge \mathop{\max }\limits_{1\le m\le M}{T}_{j}^{m}$ and 0 otherwise.

To control the FDR at the target level *q*, Knockoff-ML calculates an FDR-adaptive threshold *τ* and selects features with *W*_*j*_≥*τ*. The *τ* corresponding to the target FDR level *q* is defined as:

6 $$\tau =\min \left\{t > 0:\frac{\frac{1}{M}+\frac{1}{M}\#\{j:{\kappa }_{j}\ge 1,{\tau }_{j}\ge t\}}{\#\{j:{\kappa }_{j}=0,{\tau }_{j}\ge t\}}\le q\right\},$$

where ${\tau }_{j}={T}_{j}^{(0)}-\,\text{median}\,{T}_{j}^{(m)}$ is the largest importance score minus the median of the remaining importance scores, *κ*_*j*_ = 0 when ${T}_{j}^{0}$ is the largest importance score, and *κ*_*j*_ = *m* when ${T}_{j}^{m}$ for the *m*-th knockoff is the largest importance score. We also calculate a knockoff *q* value *q*_*j*_ for the *j*-th variable, which unifies knockoff statistic *W*_*j*_ and *τ* for variable selection. We define the knockoff *q* value for the *j*-th variable as

7 $${q}_{j}=\mathop{\min }\limits_{t\le {\tau }_{j}}\frac{\frac{1}{M}+\frac{1}{M}\#\{{j}^{{\prime} }:{\kappa }_{{j}^{{\prime} }}\ge 1,{\tau }_{{j}^{{\prime} }}\ge t\}}{\#\{{j}^{{\prime} }:{\kappa }_{{j}^{{\prime} }}=0,{\tau }_{{j}^{{\prime} }}\ge t\}}.$$

We note that selecting features with *W*_*j*_ ≥ *τ* is equivalent to selecting features with *q*_*j*_≤*q*, where *q* is the target FDR level.

### Power and FDR simulations

We implement simulations to evaluate the statistical power of Knockoff-ML in identifying risk features while preserving the FDR control. For a simulation replicate, each subject in the *n* × *p* design matrix ***X*** was simulated from a multivariate Gaussian distribution with a mean of **0** and a *p* × *p* covariance matrix **Σ** with *n* = 10,000 and *p* = 88. To mimic the real data analysis, we used the same number of variables (i.e., *p* = 88) with an empirical covariance matrix calculated from the real data as the covariance matrix **Σ** for the multivariate Gaussian distribution to simulate data. The order of variables in the simulation data aligns with that in the real data. We further dichotomized variables corresponding to binary variables in the real data, with non-negative values coded as one and negative values coded as zero. We randomly selected four features as risk features. If a risk feature is a vital sign or laboratory test that has multiple related sub-features (e.g., sbp_min, sbp_max, and sbp_mean), we included all its sub-features as risk features. For simulating linear effects of risk features on ICU mortality, we generated dichotomous traits using a logit model:

8 $$\,\text{logit}\,(E({Y}_{i}))={\beta }_{0}+{\beta }_{1}f({S}_{i1})+\cdots +{\beta }_{j}f({S}_{ij}),$$

and quantitative traits using a linear model:

9 $${Y}_{i}={\beta }_{1}f({S}_{i1})+\cdots +{\beta }_{j}f({S}_{ij})+{\epsilon }_{i},$$

where ${\epsilon }_{i} \sim {\mathscr{N}}(0,1)$, *β*_0_ was set such that the baseline ICU mortality rate was 16.2%^68^, *S*_*i*1_, …, *S*_*ij*_ were risk features, and $\,\text{logit}\,(x)=\log (\frac{x}{1-x})$. To mimic the real data with features of positive and negative effects on ICU mortality, we set *β*_1_ = − 1.5 and *β*_2_, …, *β*_*j*_ = 1.5. For simulating linear effects of risk features on ICU mortality, we defined *f*(*x*) = *x* for both traits. For simulating nonlinear effects of risk features on ICU mortality, we considered both nonlinear quadratic and exponential effects by defining *f*(*x*) = *x*^2^ for nonlinear quadratic effects and $f(x)=\exp (x)$ for nonlinear exponential effects. For each replicate, the power is defined as the proportion of identified features among all risk features, and the FDR is defined as the proportion of non-risk features among all identified features. We simulated 100 replicates and calculated the average power and FDR.

### Machine learning models for outcome prediction

We trained five ML models, including CatBoost, LightGBM, XGBoost, GBDT, and RF, to predict the risk of ICU mortality with features identified by Knockoff-ML. We partitioned data using the stratified train-test split method that allocated 70% of the data for training and the remaining 30% for testing. The stratified split method ensured that the proportion of cases and controls was consistent across both sets. To optimize the ML models, the hyperparameters for each model were obtained by a grid search combined with five rounds of five-fold cross-validation. Specifically, we divided the training set into five equally sized folds for five-fold cross-validation, using four folds for training and the remaining one fold for validation. We optimized “iterations”, “depth”, and “learning_rate” for CatBoost, LightGBM, and XGBoost, “n_estimators”, “max_depth” and “learning_rate” for GBDT, and “n_estimators”, “max_depth” and “min_samples_split” for RF. Finally, the ML models were refitted on the training set with the optimized hyperparameters.

To compare the prediction performance of Knockoff-ML with existing feature selection methods, we implemented four conventional methods, including stepwise regression, backward elimination, forward selection, and lasso. For stepwise regression, backward elimination, and forward selection, we selected the optimal model based on the Akaike information criterion and selected features that remained in the final models. For lasso, we used a 10-fold cross-validation to determine two different values of the regularization parameter *λ* (i.e., ${\lambda }_{\min }$, the value of *λ* that gives the minimum mean cross-validation error, and *λ*_1se_, the value of *λ* that gives the most regularized model such that the cross-validation error is within one standard error of the minimum) and selected features with a non-zero coefficient estimate (i.e., $\hat{\beta }\,\ne \, 0$) from lasso models trained using the two *λ*’s, respectively. We then used the same approach to train the ML models as above, with features identified by these conventional feature selection models, respectively.

The prediction performance of ML models was evaluated with several commonly used evaluation metrics, including AUROC, sensitivity, specificity, and F1 score. The DeLong test^53^ was employed to assess whether there was a significant difference between the AUROCs of different prediction models. DCA was used to evaluate the net benefit of the models at different threshold probabilities. We reported the above evaluation indicators and selected the best prediction model based on the performance of these metrics in the test set.

## Supplementary information

Supplementary information

## Data Availability

The MIMIC-IV dataset analysed during the current study is available in the PhysioNet repository, https://physionet.org.

## Code availability

The underlying code for this study is available in GitHub and can be accessed via this link https://github.com/keiwong493/Knockoff-ML.
