# Supplementary material for: Knockoff-ML: a knockoff machine learning framework for controlled variable selection and risk stratification in electronic health record data
Source: NPJ Digit Med. 2025 Nov 26;8:723. doi: 10.1038/s41746-025-02102-2 (PMC12657878; doi:10.1038/s41746-025-02102-2)
Supplement: Supplementary file 1 — Supplementary information [file 41746_2025_2102_MOESM1_ESM.pdf]

Supplementary information

Supplementary Tables

- **Supplementary Table 1.** Area under receiver operating characteristic curve (AUROC) for models with all features and features identified by Knockoff-ML in simulations with dichotomous traits.
- **Supplementary Table 2.** R-squared (R^2) for models with all features and features identified by Knockoff-ML in simulations with quantitative traits.
- **Supplementary Table 3.** Root mean square error (RMSE) for models with all features and features identified by Knockoff-ML in simulations with quantitative traits.
- **Supplementary Table 4.** Prediction performances of CatBoost with all features and features identified by different feature selection methods in simulations.
- **Supplementary Table 5.** Statistical significance of identified features for 7-day mortality using Knockoff-ML.
- **Supplementary Table 6.** Statistical significance of identified features for 30-day mortality using Knockoff-ML.
- **Supplementary Table 7.** Statistical significance of identified features for 1-year mortality using Knockoff-ML.
- **Supplementary Table 8.** Baseline characteristics of identified risk features for 7-day mortality.
- **Supplementary Table 9.** Baseline characteristics of identified risk features for 30-day mortality.
- **Supplementary Table 10.** Baseline characteristics of identified risk features for 1-year mortality.
- **Supplementary Table 11.** Baseline demographics of training and test sets for 7-day mortality.
- **Supplementary Table 12.** Baseline demographics of training and test sets for 30-day mortality.
- **Supplementary Table 13.** Baseline demographics of training and test sets for 1-year mortality.
- **Supplementary Table 14.** Performance of lasso with λ_{min} and λ_{1se} in simulation studies.

Supplementary Figures

- **Supplementary Fig. 1.** Power and FDR of Knockoff-ML and conventional feature selection methods in simulation studies.
- **Supplementary Fig. 2.** Number of features identified by Knockoff-ML and conventional feature selection methods.
- **Supplementary Fig. 3.** Risk features identified by Knockoff-ML.
- **Supplementary Fig. 4.** Significance of difference in area under receiver operating characteristic curve (AUROC) between different predcition models.
- **Supplementary Fig. 5.** Area under receiver operating characteristic curve (AUROC) for models using Knockoff-ML's lowest-ranked features.
- **Supplementary Fig. 6.** Area under receiver operating characteristic curve (AUROC) for CatBoost using features identified by Knockoff-ML and conventional feature selection methods.
- **Supplementary Fig. 7.** Area under receiver operating characteristic curve (AUROC) for LightGBM using features identified by Knockoff-ML and conventional feature selection methods.
- **Supplementary Fig. 8.** Area under receiver operating characteristic curve (AUROC) for XGBoost using features identified by Knockoff-ML and conventional feature selection methods.

- **Supplementary Fig. 9.** Area under receiver operating characteristic curve (AUROC) for GBDT using features identified by Knockoff-ML and conventional feature selection methods.
- **Supplementary Fig. 10.** Area under receiver operating characteristic curve (AUROC) for RF using features identified by Knockoff-ML and conventional feature selection methods.
- **Supplementary Fig. 11.** Distribution of predicted probabilities from final prediction models based on risk features identified by Knockoff-ML for mortality outcomes.
- **Supplementary Fig. 12.** Heatmap of feature correlations in the MIMIC-IV dataset.
- **Supplementary Fig. 13.** Mean cross-validation error in lasso with different λ values.

Supplementary Tables

Supplementary Table 1: Area under receiver operating characteristic curve (AUROC) for models with all features and features identified by Knockoff-ML in simulations with dichotomous traits.

Model	Linear effects		Nonlinear quadratic		Nonlinear exponential	
	Full	Knockoff-ML	Full	Knockoff-ML	Full	Knockoff-ML
CatBoost	0.998	0.998	0.995	0.996	0.998	0.997
LightGBM	0.996	0.996	0.993	0.994	0.997	0.996
XGBoost	0.996	0.997	0.992	0.995	0.997	0.996
GBDT	0.995	0.994	0.988	0.985	0.996	0.995
RF	0.978	0.974	0.967	0.965	0.983	0.980

"Full" indicates that models were trained with all features, while "Knockoff-ML" indicates that models were trained with features identified by Knockoff-ML. The AUROC was averaged over 100 replicates. CatBoost: categorical boosting; LightGBM: light gradient boosting machine; XGBoost: eXtreme gradient boosting; GBDT: gradient boosting decision tree; RF: random forest.

Supplementary Table 2: R-squared (R^2) for models with all features and features identified by Knockoff-ML in simulations with quantitative traits.

Model	Linear effects		Nonlinear quadratic		Nonlinear exponential	
	Full	Knockoff-ML	Full	Knockoff-ML	Full	Knockoff-ML
CatBoost	0.890	0.843	0.904	0.848	0.939	0.874
LightGBM	0.884	0.830	0.894	0.837	0.937	0.867
XGBoost	0.864	0.806	0.870	0.822	0.917	0.830
GBDT	0.886	0.830	0.894	0.835	0.950	0.867
RF	0.858	0.792	0.859	0.800	0.931	0.852

"Full" indicates that models were trained with all features, while "Knockoff-ML" indicates that models were trained with features identified by Knockoff-ML. The R^2 was averaged over 100 replicates. CatBoost: categorical boosting; LightGBM: light gradient boosting machine; XGBoost: eXtreme gradient boosting; GBDT: gradient boosting decision tree; RF: random forest.

Supplementary Table 3: Root mean square error (RMSE) for models with all features and features identified by Knockoff-ML in simulations with quantitative traits.

Model	Linear effects		Nonlinear quadratic		Nonlinear exponential	
	Full	Knockoff-ML	Full	Knockoff-ML	Full	Knockoff-ML
CatBoost	1.053	1.347	1.231	1.647	1.767	2.737
LightGBM	1.103	1.412	1.339	1.739	2.135	3.092
XGBoost	1.216	1.535	1.505	1.828	2.053	2.951
GBDT	1.100	1.405	1.325	1.745	2.376	3.358
RF	1.283	1.585	1.619	1.958	2.005	2.873

"Full" indicates that models were trained with all features, while "Knockoff-ML" indicates that models were trained with features identified by Knockoff-ML. The RMSE was averaged over 100 replicates. CatBoost: categorical boosting; LightGBM: light gradient boosting machine; XGBoost: eXtreme gradient boosting; GBDT: gradient boosting decision tree; RF: random forest.

Supplementary Table 4: Prediction performance of CatBoost with all features and features identified by different feature selection methods in simulations.

Method	Dichotomous (AUROC)		
	Linear effects	Nonlinear quadratic	Nonlinear exponential
Full	0.9977	0.9947	0.9981
Knockoff-ML	0.9984	0.9960	0.9971
Stepwsie	0.9985	0.8354	0.9984
Backward	0.9985	0.8320	0.9984
Forward	0.9985	0.8088	0.9985
Lasso-min	0.9977	0.7989	0.9983
Lasso-1se	0.9977	0.7350	0.9985
**Method**	**Quantitative (RMSE)**		
	**Linear effects**	**Nonlinear quadratic**	**Nonlinear exponential**
Full	1.0532	1.2312	1.7672
Knockoff-ML	1.3470	1.6472	2.8742
Stepwsie	1.0477	3.3298	2.0037
Backward	1.0473	3.3295	2.0019
Forward	1.0457	3.5559	1.9947
Lasso-min	1.0480	3.8579	2.0035
Lasso-1se	1.0386	4.0904	2.2464
**Method**	**Quantitative (R^2)**		
	**Linear effects**	**Nonlinear quadratic**	**Nonlinear exponential**
Full	0.8905	0.9035	0.9394
Knockoff-ML	0.8434	0.8477	0.8735
Stepwsie	0.8912	0.4882	0.9395
Backward	0.8912	0.4883	0.9397
Forward	0.8915	0.4243	0.9402
Lasso-min	0.8911	0.3366	0.9395
Lasso-1se	0.8937	0.1718	0.9246

"Full" indicates that CatBoost models were trained with all features. "Knockoff-ML" indicates that CatBoost models were trained with features identified by Knockoff-ML with CatBoost. "Stepwise" indicates that CatBoost models were trained with features identified by stepwise regression. "Backward" indicates that CatBoost models were trained with features identified by backward elimination. "Forward" indicates that CatBoost models were trained with features identified by forward selection. 'Lasso-min" indicates that CatBoost models were trained with features identified by lasso with the regularization parameter $\lambda_{\min}$ that gives the minimum mean cross-validation error. "Lasso-1se" indicates that CatBoost models were trained with features identified by lasso with the regularization parameter λ_{1se} that gives the most regularized model such that the cross-validation error is within one standard error of the minimum. The AUROC, RMSE, and R^2 were averaged over 100 replicates. RMSE: root mean squared error; R^2: R-squared; lasso: least absolute shrinkage and selection operator.

Supplementary Table 5: Statistical significance of identified features for 7-day mortality using Knockoff-ML.

Variable	CatBoost	LightGBM	XGBoost	GBDT	RF
Age	0.0143	0.0154	0.0154	0.0333	0.0125
Heart rate (min)	0.0143	0.0154	0.0154	0.0714	0.0125
Heart rate (max)	0.0143	0.0154	-	0.0667	0.0125
Heart rate (mean)	0.0143	0.0222	0.0154	0.0444	0.0125
Temperature (max)	0.0143	0.0154	0.0154	0.0444	0.0125
Temperature (mean)	-	-	-	-	0.0125
Spo2 (min)	0.0143	0.0154	0.0154	0.0333	0.0125
Hematocrit (min)	0.0143	0.0222	0.0154	-	0.0941
Platelets (min)	-	-	-	-	0.0125
Platelets max	0.0632	0.0316	0.0353	0.0600	0.0125
WBC (min)	0.0143	0.0154	0.0154	0.0333	0.0125
WBC (max)	0.0143	0.0222	0.0353	-	0.0125
BUN (min)	-	0.0222	0.0154	-	0.0125
Glucose (min)	-	-	-	-	0.0125
Glucose (max)	0.0143	0.0154	0.0154	0.0333	0.0125
Urine output	0.0143	0.0154	0.0154	0.0333	0.0125
Cerebrovascular disease	0.0143	0.0154	0.0154	0.0333	0.0125
SBP (min)	0.0267	0.0154	-	0.0714	-
Resp rate (min)	0.0143	0.0154	0.0154	0.0444	-
Anion gap (min)	-	0.0154	0.0353	0.0667	-
Admission type Medical	0.0353	0.0154	-	0.0933	-
BUN (max)	0.0143	-	-	-	-
Congestive heart failure	0.0632	-	-	-	-
Diabetes without cc	0.0353	0.0222	-	-	-
DBP (mean)	-	-	0.0286	-	-
Hemoglobin (max)	-	-	0.0154	-	-
Calcium (min)	-	-	0.0889	-	-

Knockoff q values were listed in the table and "-" indicated that variables were not identified by Knockoff-ML. "Max", "Mean", and "Min" indicate the maximum, mean, and minimum values of a risk feature, respectively, within 24 hours after ICU admission. CatBoost: categorical boosting; LightGBM: light gradient boosting machine; XGBoost: eXtreme gradient boosting; GBDT: gradient boosting decision tree; RF: random forest; WBC: white blood cell; BUN: blood urea nitrogen; SBP: systolic blood pressure; DBP: diastolic blood pressure; Resp rate: respiratory rate.

Supplementary Table 6: Statistical significance of identified features for 30-day mortality using Knockoff-ML.

Variable	CatBoost	LightGBM	XGBoost	GBDT	RF
Age	0.0125	0.0182	0.0154	0.0182	0.0125
Heart rate (min)	0.0125	0.0267	0.0636	-	0.0125
Heart rate (max)	0.0125	0.0182	0.0353	0.0182	0.0125
Heart rate (mean)	0.0125	0.0182	0.0154	0.0286	0.0125
Temperature (max)	0.0125	0.0182	0.0154	0.0182	0.0125
Temperature (mean)	-	-	-	-	0.0125
Spo2 (min)	0.0125	0.0182	0.0154	0.0182	0.0125
Platelets (min)	-	-	-	0.0933	0.0125
Platelets max	0.0421	0.0267	-	0.0286	0.0125
WBC (min)	0.0125	0.0182	0.0154	0.0182	0.0125
WBC max	-	-	-	-	0.0125
BUN (min)	0.0125	0.0182	0.0154	0.0182	0.0125
Glucose (min)	-	0.0267	0.0353	-	0.0125
Glucose (max)	0.0125	0.0182	0.0154	0.0182	0.0125
Potassium (min)	0.0222	-	-	-	0.0700
PT (min)	-	0.0737	-	-	0.0222
PT (max)	-	-	-	-	0.0700
PTT (min)	-	-	-	-	0.0762
Urine output	0.0125	0.0182	0.0154	0.0182	0.0125
Cerebrovascular disease	0.0125	0.0182	0.0154	0.0182	0.0125
Metastatic solid tumor	0.0125	0.0471	0.0154	0.0182	0.0222
Mild liver disease	-	0.0375	-	0.0286	-
Admission type Medical	0.0125	0.0182	0.0154	0.0182	-
Gender	0.0455	-	0.0421	-	-
Hematocrit (min)	0.0125	0.0667	0.0154	-	-
Hemoglobin (min)	0.0222	-	0.0353	-	-
Hemoglobin (max)	0.0455	0.0267	0.0154	-	-
BUB (max)	0.0125	-	-	-	-
INR (max)	0.0125	-	-	-	-
Diabetes with comorbidity	0.0455	-	-	-	-
SBP (min)	-	-	0.0636	-	-
DBP (mean)	-	-	0.0696	-	-
MBP (mean)	-	-	0.0421	-	-
Resp rate (min)	-	-	0.0636	-	-
Hematocrit (max)	-	-	0.0353	-	-

Knockoff q values were listed in the table and "-" indicated that variables were not identified by Knockoff-ML. "Max", "Mean", and "Min" indicate the maximum, mean, and minimum values of a risk feature, respectively, within 24 hours after ICU admission. CatBoost: categorical boosting; LightGBM: light gradient boosting machine; XGBoost: eXtreme gradient boosting; GBDT: gradient boosting decision tree; RF: random forest; WBC: white blood cell; BUN: blood urea nitrogen; SBP: systolic blood pressure; DBP: diastolic blood pressure; Resp rate: respiratory rate; PT: prothrombin time; PTT: partial thromboplastin time; INR: international normalized ratio; MBP: mean blood pressure.

Supplementary Table 7: Statistical significance of identified features for 1-year mortality using Knockoff-ML.

Variable	CatBoost	LightGBM	XGBoost	GBDT	RF
Age	0.0143	0.0143	0.0133	0.0200	0.0111
Heart rate (min)	0.0667	-	0.0526	-	0.0833
Heart rate (max)	0.0143	0.0143	0.0333	0.0200	0.0111
Heart rate (mean)	0.0526	0.0588	0.0333	-	0.0111
Temperature (max)	0.0143	0.0143	0.0133	0.0941	0.0111
Temperature (mean)	-	-	-	-	0.0111
Spo2 (min)	0.0143	0.0143	0.0133	0.0200	0.0111
Hematocrit (max)	-	-	0.0133	-	0.0526
Hemoglobin (min)	-	-	0.0133	-	0.0818
Hemoglobin (max)	0.0143	0.0143	0.0133	0.0941	0.0111
Platelets (min)	-	-	-	0.0941	0.0111
Platelets (max)	0.0250	0.0143	0.0333	0.0545	0.0111
WBC (min)	0.0143	0.0143	0.0133	0.0200	0.0111
WBC (max)	-	-	-	-	0.0111
BUN (min)	0.0143	0.0143	0.0133	0.0200	0.0111
BUN (max)	-	-	-	-	0.0600
Glucose (min)	0.0143	0.0143	0.0133	0.0941	0.0111
Glucose (max)	0.0143	0.0143	0.0133	0.0200	0.0111
Potassium (min)	0.0471	-	0.0700	-	0.0762
PTT (min)	-	-	-	-	0.0833
Urine output	0.0143	0.0143	0.0133	0.0200	0.0111
Cerebrovascular disease	0.0143	0.0143	0.0133	0.0200	0.0111
Malignant cancer	0.0143	0.0143	0.0133	0.0200	0.0111
Metastatic solid tumor	-	-	-	-	0.0111
INR (min)	-	-	-	0.1000	-
Mild liver disease	0.0526	0.0143	0.0909	0.0200	-
Insurance Other	-	0.0250	-	0.0833	-
Admission type Medical	0.0250	0.1000	-	0.0941	-
Gender	0.0143	0.0250	0.0133	-	-
Hematocrit (min)	0.0143	0.1000	0.0133	-	-
Potassium (max)	0.0667	-	0.0909	-	-
Creatinine (min)	-	0.1000	-	-	-

Knockoff q values were listed in the table and "-" indicated that variables were not identified by Knockoff-ML. "Max", "Mean", and "Min" indicate the maximum, mean, and minimum values of a risk feature, respectively, within 24 hours after ICU admission. CatBoost: categorical boosting; LightGBM: light gradient boosting machine; XGBoost: eXtreme gradient boosting; GBDT: gradient boosting decision tree; RF: random forest; WBC: white blood cell; BUN: blood urea nitrogen; PTT: partial thromboplastin time; INR: international normalized ratio.

Supplementary Table 8: Baseline characteristics of identified risk features for 7-day mortality.

	ALL (N=50,921)	7-day Survival (N=47,148)	7-day Mortality (N=3,773)	p value
Age	64.9 (17.3)	64.3 (17.2)	72.4 (16.0)	4.89×10^{-174}
Urine output	1,794 (1,234)	1,840 (1,205)	1,194 (1,440)	9.91×10^{-136}
Cerebrovascular disease				2.25×10^{-50}
No	42,517 (83.5%)	39,713 (84.2%)	2,804 (74.3%)	
Yes	8,404 (16.5%)	7,435 (15.8%)	969 (25.7%)	
Heart rate (mean)	84.3 (15.7)	83.7 (15.3)	90.8 (19.3)	4.48×10^{-100}
Hematocrit (min)	31.3 (6.70)	31.3 (6.64)	30.9 (7.36)	1.46×10^{-03}
Temperature (max)	37.3 (0.72)	37.3 (0.66)	37.2 (1.23)	1.55×10^{-10}
WBC (min)	10.4 (8.02)	10.2 (7.10)	13.1 (15.2)	1.26×10^{-31}
BUN (min)	21.9 (18.4)	21.0 (17.4)	34.0 (25.8)	4.74×10^{-179}
SpO2 (min)	91.8 (6.59)	92.3 (5.08)	85.8 (15.1)	2.42×10^{-139}
Respiratory rate (min)	12.3 (3.55)	12.2 (3.42)	13.8 (4.73)	3.28×10^{-84}
Glucose (max)	164 (99.8)	160 (96.6)	205 (127)	1.70×10^{-91}
Heart rate (min)	69.9 (15.1)	69.8 (14.5)	71.6 (21.3)	2.66×10^{-07}
Heart rate (max)	102 (20.5)	102 (19.9)	112 (24.5)	9.90×10^{-127}
WBC (max)	14.0 (11.6)	13.7 (10.7)	17.7 (19.4)	1.77×10^{-34}
Anion gap (min)	13.0 (3.52)	12.8 (3.20)	16.1 (5.47)	6.79×10^{-244}
SBP (min)	93.5 (17.5)	94.3 (16.6)	82.5 (24.0)	4.28×10^{-173}
Platelets (max)	227 (109)	228 (108)	219 (124)	9.69×10^{-05}
Admission type				1.00×10^{-04}
Medical	35,133 (69.0%)	31,974 (67.8%)	3,159 (83.7%)	
Scheduled	1,537 (3.02%)	1,529 (3.24%)	8 (0.21%)	
Unscheduled	14,251 (28.0%)	13,645 (28.9%)	606 (16.1%)	

Mean and standard deviation were reported for continuous variables, while counts and percentages were reported for categorical variables. P values are obtained from t test for continuous variables and Fisher's exact test for categorical variables. "Max", "Mean", and "Min" indicate the maximum, mean, and minimum values of a risk feature, respectively, within 24 hours after ICU admission. WBC: white blood cell; BUN: blood urea nitrogen; SBP: systolic blood pressure.

Supplementary Table 9: Baseline characteristics of identified risk features for 30-day mortality.

	**ALL N=50,921**	**30-day Survival N=44,192**	**30-day Mortality N=6,729**	**p value**
Age	64.9 (17.3)	63.8 (17.2)	72.7 (15.3)	$< 0.00 \times 10^{-300}$
Urine output	1,794 (1,234)	1,872 (1,209)	1,271 (1,273)	4.11×10^{-254}
Cerebrovascular disease				1.15×10^{-66}
No	42,517 (83.5%)	37,410 (84.7%)	5,107 (75.9%)	
Yes	8,404 (16.5%)	6,782 (15.3%)	1,622 (24.1%)	
BUN (min)	21.9 (18.4)	20.2 (16.6)	33.0 (24.9)	1.98×10^{-323}
WBC (min)	10.4 (8.02)	10.0 (6.81)	12.6 (13.3)	2.05×10^{-52}
Temperature (max)	37.3 (0.72)	37.3 (0.65)	37.3 (1.04)	1.86×10^{-09}
Hematocrit (min)	31.3 (6.70)	31.4 (6.63)	30.3 (7.05)	3.28×10^{-30}
Hemoglobin (max)	11.7 (2.13)	11.8 (2.10)	11.3 (2.28)	1.98×10^{-68}
Glucose (max)	164 (99.8)	159 (96.5)	192 (116)	2.81×10^{-98}
SpO2 (min)	91.8 (6.59)	92.4 (4.93)	88.0 (12.4)	7.79×10^{-169}
Heart rate (mean)	84.3 (15.7)	83.5 (15.2)	89.5 (18.2)	6.38×10^{-142}
Heart rate (max)	102 (20.5)	101 (19.8)	110 (23.4)	2.28×10^{-168}
Admission type				1.00×10^{-04}
Medical	35,133 (69.0%)	29,508 (66.8%)	5,625 (83.6%)	
Scheduled surgical	1,537 (3.02%)	1,509 (3.41%)	28 (0.42%)	
Unscheduled surgical	14,251 (28.0%)	13,175 (29.8%)	1,076 (16.0%)	
Metastatic solid tumor				8.02×10^{-198}
No	47,766 (93.8%)	42,084 (95.2%)	5,682 (84.4%)	
Yes	3,155 (6.20%)	2,108 (4.77%)	1,047 (15.6%)	
Heart rate (min)	69.9 (15.1)	69.5 (14.4)	72.3 (19.1)	8.62×10^{-31}
Glucose (min)	119 (41.1)	118 (37.8)	127 (57.7)	2.76×10^{-34}
Platelets (max)	227 (109)	227 (106)	225 (129)	1.54×10^{-01}

Mean and standard deviation were reported for continuous variables, while counts and percentages were reported for categorical variables. P values are obtained from t test for continuous variables and Fisher's exact test for categorical variables. "Max", "Mean", and "Min" indicate the maximum, mean, and minimum values of a risk feature, respectively, within 24 hours after ICU admission. WBC: white blood cell; BUN: blood urea nitrogen.

Supplementary Table 10: Baseline characteristics of identified risk features for 1-year mortality.

	ALL N=50,921	1-year Survival N=38,385	1-year Mortality N=12,536	p value
Age	64.9 (17.3)	62.4 (17.3)	72.6 (14.8)	$< 0.00 \times 10^{-300}$
BUN (min)	21.9 (18.4)	19.0 (15.3)	30.8 (23.7)	$< 0.00 \times 10^{-300}$
Urine output	1,794 (1234)	1,915 (1214)	1,413 (1218)	$< 0.00 \times 10^{-300}$
Hemoglobin max	11.7 (2.13)	11.9 (2.08)	11.1 (2.18)	4.20×10^{-264}
Cerebrovascular disease				2.94×10^{-36}
No	42,517 (83.5%)	32,512 (84.7%)	10,005 (79.8%)	
Yes	8,404 (16.5%)	5,873 (15.3%)	2,531 (20.2%)	
Malignant cancer				$< 0.00 \times 10^{-300}$
No	44,465 (87.3%)	35,225 (91.8%)	9,240 (73.7%)	
Yes	6,456 (12.7%)	3,160 (8.23%)	3,296 (26.3%)	
Hematocrit (min)	31.3 (6.70)	31.6 (6.63)	30.1 (6.80)	4.73×10^{-98}
SpO2 (min)	91.8 (6.59)	92.5 (4.81)	89.7 (9.99)	1.06×10^{-192}
Glucose (max)	164 (99.8)	158 (95.8)	180 (109)	6.80×10^{-90}
WBC (min)	10.4 (8.02)	9.99 (6.47)	11.5 (11.5)	7.03×10^{-46}
Glucose (min)	119 (41.1)	118 (37.2)	123 (51.1)	1.29×10^{-19}
Gender				5.69×10^{-08}
Female	22,480 (44.1%)	16,683 (43.5%)	5,797 (46.2%)	
Male	28,441 (55.9%)	21,702 (56.5%)	6,739 (53.8%)	
Temperature (max)	37.3 (0.72)	37.4 (0.65)	37.3 (0.88)	2.43×10^{-25}
Heart rate (max)	102 (20.5)	101 (19.5)	107 (22.7)	5.33×10^{-130}
Heart rate (mean)	84.3 (15.7)	83.3 (15.1)	87.2 (17.2)	9.06×10^{-108}
Platelets (max)	227 (109)	227 (102)	228 (127)	3.00×10^{-01}
Mild liver disease				3.70×10^{-112}
No	45,683 (89.7%)	35,130 (91.5%)	10,553 (84.2%)	
Yes	5,238 (10.3%)	3,255 (8.48%)	1,983 (15.8%)	
Admission type				1.00×10^{-04}
Medical	35,133 (69.0%)	24,945 (65.0%)	10,188 (81.3%)	
Scheduled surgical	1,537 (3.02%)	1,439 (3.75%)	98 (0.78%)	
Unscheduled surgical	14,251 (28.0%)	12,001 (31.3%)	2,250 (17.9%)	
Heart rate min	69.9 (15.1)	69.4 (14.3)	71.6 (17.3)	5.74×10^{-38}
Potassium min	3.92 (0.56)	3.91 (0.52)	3.96 (0.67)	7.34×10^{-13}

Mean and standard deviation were reported for continuous variables, while counts and percentages were reported for categorical variables. P values are obtained from t test for continuous variables and Fisher's exact test for categorical variables. "Max", "Mean", and "Min" indicate the maximum, mean, and minimum values of a risk feature, respectively, within 24 hours after ICU admission. WBC: white blood cell; BUN: blood urea nitrogen.

Supplementary Table 11: Baseline demographics of training and test sets for 7-day mortality.

	Training set			Test set		
	**ALL N=35,644**	**7-day survival N=33,003 (92.59%)**	**7-day mortality N=2,641 (7.41%)**	**ALL N=15,277**	**7-day survival N=14,145 (92.59%)**	**7-day mortality N=1,132 (7.41%)**
Age	64.9 (17.2)	64.3 (17.2)	72.2 (15.9)	65.1 (17.4)	64.5 (17.3)	72.6 (16.2)
Gender						
Female	15,823 (44.4%)	14,546 (44.1%)	1,277 (48.4%)	6,657 (43.6%)	6,134 (43.4%)	523 (46.2%)
Male	19,821 (55.6%)	18,457 (55.9%)	1,364 (51.6%)	8,620 (56.4%)	8,011 (56.6%)	609 (53.8%)
Race						
White	23,899 (67.0%)	22,345 (67.7%)	1,554 (58.8%)	10,306 (67.5%)	9,629 (68.1%)	677 (59.8%)
Asian	1,133 (3.18%)	1,038 (3.15%)	95 (3.60%)	444 (2.91%)	411 (2.91%)	33 (2.92%)
Black	3,260 (9.15%)	3,076 (9.32%)	184 (6.97%)	1,380 (9.03%)	1,304 (9.22%)	76 (6.71%)
Hispanic	1,216 (3.41%)	1,150 (3.48%)	66 (2.50%)	519 (3.40%)	500 (3.53%)	19 (1.68%)
Other	6,136 (17.2%)	5,394 (16.3%)	742 (28.1%)	2,628 (17.2%)	2,301 (16.3%)	327 (28.9%)
Marital status						
Married	16,193 (45.4%)	15,199 (46.1%)	994 (37.6%)	6,911 (45.2%)	6,465 (45.7%)	446 (39.4%)
Single	9,628 (27.0%)	9,104 (27.6%)	524 (19.8%)	4,013 (26.3%)	3,835 (27.1%)	178 (15.7%)
Divorced	2,486 (6.97%)	2,341 (7.09%)	145 (5.49%)	1,088 (7.12%)	1,038 (7.34%)	50 (4.42%)
Widowed	4,266 (12.0%)	3,816 (11.6%)	450 (17.0%)	1,910 (12.5%)	1,694 (12.0%)	216 (19.1%)
Missing	3,071 (8.62%)	2,543 (7.71%)	528 (20.0%)	1,355 (8.87%)	1,113 (7.87%)	242 (21.4%)
Insurance type						
Medicaid	2,529 (7.10%)	2,395 (7.26%)	134 (5.07%)	1,111 (7.27%)	1,030 (7.28%)	81 (7.16%)
Medicare	15,239 (42.8%)	13,811 (41.8%)	1,428 (54.1%)	6,563 (43.0%)	5,959 (42.1%)	604 (53.4%)
Other	17,876 (50.2%)	16,797 (50.9%)	1,079 (40.9%)	7,603 (49.8%)	7,156 (50.6%)	447 (39.5%)
Admission type						
Medical	24,602 (69.0%)	22,394 (67.9%)	2,208 (83.6%)	10,531 (68.9%)	9,580 (67.7%)	951 (84.0%)
Scheduled	1,050 (2.95%)	1,045 (3.17%)	5 (0.19%)	487 (3.19%)	484 (3.42%)	3 (0.27%)
Unscheduled	9,992 (28.0%)	9,564 (29.0%)	428 (16.2%)	4,259 (27.9%)	4,081 (28.9%)	178 (15.7%)
SOFA	4.22 (3.30)	3.93 (3.01)	7.77 (4.51)	4.26 (3.32)	3.97 (3.02)	7.88 (4.55)
SAPS II	34.0 (13.9)	32.7 (12.7)	51.1 (16.7)	34.2 (14.0)	32.9 (12.8)	51.5 (16.6)

Mean and standard deviation were reported for continuous variables, while counts and percentages were reported for categorical variables. SOFA: sequential organ failure assessment; SAPS II: simplified acute physiology score II.

Supplementary Table 12: Baseline demographics of training and test sets for 30-day mortality.

	Training set			Test set		
	**ALL N=35,644**	**30-day survival N=30,934 (86.79%)**	**30-day mortality N=4,710 (13.21%)**	**ALL N=15,277**	**30-day survival N=13,258 (86.78%)**	**30-day mortality N=2,019 (13.22%)**
Age	64.9 (17.3)	63.7 (17.3)	72.7 (15.4)	65.0 (17.3)	63.8 (17.2)	72.7 (15.3)
Gender						
Female	15,774 (44.3%)	13,536 (43.8%)	2,238 (47.5%)	6,706 (43.9%)	5,771 (43.5%)	935 (46.3%)
Male	19,870 (55.7%)	17,398 (56.2%)	2,472 (52.5%)	8,571 (56.1%)	7,487 (56.5%)	1,084 (53.7%)
Race						
White	23,911 (67.1%)	20,956 (67.7%)	2,955 (62.7%)	10,294 (67.4%)	9,043 (68.2%)	1,251 (62.0%)
Asian	1,123 (3.15%)	967 (3.13%)	156 (3.31%)	454 (2.97%)	392 (2.96%)	62 (3.07%)
Black	3,291 (9.23%)	2,938 (9.50%)	353 (7.49%)	1,349 (8.83%)	1,206 (9.10%)	143 (7.08%)
Hispanic	1,244 (3.49%)	1,152 (3.72%)	92 (1.95%)	491 (3.21%)	444 (3.35%)	47 (2.33%)
Other	6,075 (17.0%)	4,921 (15.9%)	1,154 (24.5%)	2,689 (17.6%)	2,173 (16.4%)	516 (25.6%)
Marital status						
Married	16,230 (45.5%)	14,383 (46.5%)	1,847 (39.2%)	6,874 (45.0%)	6,077 (45.8%)	797 (39.5%)
Single	9,515 (26.7%)	8,597 (27.8%)	918 (19.5%)	4,126 (27.0%)	3,724 (28.1%)	402 (19.9%)
Divorced	2,507 (7.03%)	2,223 (7.19%)	284 (6.03%)	1,067 (6.98%)	950 (7.17%)	117 (5.79%)
Widowed	4,340 (12.2%)	3,486 (11.3%)	854 (18.1%)	1,836 (12.0%)	1,476 (11.1%)	360 (17.8%)
Missing	3,052 (8.56%)	2,245 (7.26%)	807 (17.1%)	1,374 (8.99%)	1,031 (7.78%)	343 (17.0%)
Insurance type						
Medicaid	2,535 (7.11%)	2,268 (7.33%)	267 (5.67%)	1,105 (7.23%)	989 (7.46%)	116 (5.75%)
Medicare	15,212 (42.7%)	12,659 (40.9%)	2,553 (54.2%)	6,590 (43.1%)	5,463 (41.2%)	1,127 (55.8%)
Other	17,897 (50.2%)	16,007 (51.7%)	1,890 (40.1%)	7,582 (49.6%)	6,806 (51.3%)	776 (38.4%)
Admission type						
Medical	24,584 (69.0%)	20,667 (66.8%)	3,917 (83.2%)	10,549 (69.1%)	8,841 (66.7%)	1,708 (84.6%)
Scheduled	1,065 (2.99%)	1,048 (3.39%)	17 (0.36%)	472 (3.09%)	461 (3.48%)	11 (0.54%)
Unscheduled	9,995 (28.0%)	9,219 (29.8%)	776 (16.5%)	4,256 (27.9%)	3,956 (29.8%)	300 (14.9%)
SOFA	4.22 (3.29)	3.80 (2.90)	6.94 (4.25)	4.26 (3.36)	3.83 (2.93)	7.14 (4.39)
SAPS II	34.0 (14.0)	31.9 (12.4)	48.1 (15.5)	34.2 (14.0)	32.1 (12.4)	48.2 (15.4)

Mean and standard deviation were reported for continuous variables, while counts and percentages were reported for categorical variables. SOFA: sequential organ failure assessment; SAPS II: simplified acute physiology score II.

Supplementary Table 13: Baseline demographics of training and test sets for 1-year mortality.

	**Training set**			**Test set**		
	**ALL N=35,644**	**1-year survival N=26,869 (75.38%)**	**1-year mortality N=8,775 (24.62%)**	**ALL N=15,277**	**1-year survival N=11,516 (75.38%)**	**1-year mortality N=3,761 (24.62%)**
Age	65.0 (17.3)	62.5 (17.3)	72.7 (14.8)	64.8 (17.3)	62.3 (17.3)	72.4 (14.9)
Gender						
Female	15,747 (44.2%)	11,677 (43.5%)	4,070 (46.4%)	6,733 (44.1%)	5,006 (43.5%)	1,727 (45.9%)
Male	19,897 (55.8%)	15,192 (56.5%)	4,705 (53.6%)	8,544 (55.9%)	6,510 (56.5%)	2,034 (54.1%)
Race						
White	23,910 (67.1%)	18,037 (67.1%)	5,873 (66.9%)	10,295 (67.4%)	7,753 (67.3%)	2,542 (67.6%)
Asian	1,133 (3.18%)	861 (3.20%)	272 (3.10%)	444 (2.91%)	323 (2.80%)	121 (3.22%)
Black	3,292 (9.24%)	2,541 (9.46%)	751 (8.56%)	1,348 (8.82%)	1,056 (9.17%)	292 (7.76%)
Hispanic	1,202 (3.37%)	1,012 (3.77%)	190 (2.17%)	533 (3.49%)	435 (3.78%)	98 (2.61%)
Other	6,107 (17.1%)	4,418 (16.4%)	1,689 (19.2%)	2,657 (17.4%)	1,949 (16.9%)	708 (18.8%)
Marital status						
Married	16,160 (45.3%)	12,563 (46.8%)	3,597 (41.0%)	6,944 (45.5%)	5,368 (46.6%)	1,576 (41.9%)
Single	9,640 (27.0%)	7,668 (28.5%)	1,972 (22.5%)	4,001 (26.2%)	3,227 (28.0%)	774 (20.6%)
Divorced	2,461 (6.90%)	1,875 (6.98%)	586 (6.68%)	1,113 (7.29%)	850 (7.38%)	263 (6.99%)
Widowed	4,315 (12.1%)	2,751 (10.2%)	1,564 (17.8%)	1,861 (12.2%)	1,187 (10.3%)	674 (17.9%)
Missing	3,068 (8.61%)	2,012 (7.49%)	1,056 (12.0%)	1,358 (8.89%)	884 (7.68%)	474 (12.6%)
Insurance type						
Medicaid	2,506 (7.03%)	2,025 (7.54%)	481 (5.48%)	1,134 (7.42%)	896 (7.78%)	238 (6.33%)
Medicare	15,321 (43.0%)	10,420 (38.8%)	4,901 (55.9%)	6,481 (42.4%)	4,391 (38.1%)	2,090 (55.6%)
Other	17,817 (50.0%)	14,424 (53.7%)	3,393 (38.7%)	7,662 (50.2%)	6,229 (54.1%)	1,433 (38.1%)
Admission type						
Medical	24,584 (69.0%)	17,443 (64.9%)	7,141 (81.4%)	10,549 (69.1%)	7,502 (65.1%)	3,047 (81.0%)
Scheduled	1,085 (3.04%)	1,013 (3.77%)	72 (0.82%)	452 (2.96%)	426 (3.70%)	26 (0.69%)
Unscheduled	9,975 (28.0%)	8,413 (31.3%)	1,562 (17.8%)	4,276 (28.0%)	3,588 (31.2%)	688 (18.3%)
SOFA	4.23 (3.31)	3.67 (2.85)	5.94 (3.98)	4.23 (3.29)	3.71 (2.84)	5.84 (3.99)
SAPS II	34.1 (13.9)	30.9 (12.1)	44.1 (14.5)	34.0 (14.0)	30.9 (12.1)	43.7 (14.9)

Mean and standard deviation were reported for continuous variables, while counts and percentages were reported for categorical variables. SOFA: sequential organ failure assessment; SAPS II: simplified acute physiology score II.

Supplementary Table 14: Performance of lasso with $\lambda_{\min}$ and λ_{1se} in simulation studies.

	Dichotomous traits					
	**Linear effects**		**Nonlinear quadratic**		**Nonlinear exponential**	
	$\lambda_{\min}$	λ_{1se}	$\lambda_{\min}$	λ_{1se}	$\lambda_{\min}$	λ_{1se}
Power	1	1	5.09E-01	4.20E-01	1	1
FDR	8.34E-01	8.31E-01	6.05E-01	3.10E-02	7.90E-01	6.14E-01
# of selected features	69.08	67.09	11.80	2.24	45.68	20.93
λ value	4.26E-05	5.41E-05	6.24E-03	2.05E-02	6.97E-04	2.10E-03
MCE	1.78E-02	1.92E-02	1.08E+00	1.09E+00	2.29E-01	2.35E-01
SE	1.63E-03	1.56E-03	5.52E-03	4.51E-03	6.99E-03	6.13E-03
	**Quantitative traits**					
	**Linear effects**		**Nonlinear quadratic**		**Nonlinear exponential**	
	$\lambda_{\min}$	λ_{1se}	$\lambda_{\min}$	λ_{1se}	$\lambda_{\min}$	λ_{1se}
Power	1	1	5.21E-01	4.47E-01	1	9.38E-01
FDR	7.02E-01	1.26E-01	5.64E-01	0	6.88E-01	1.77E-02
# of selected features	25.23	8.00	9.62	1.96	23.97	5.97
λ value	1.08E-02	4.51E-02	7.88E-02	5.13E-01	5.45E-02	6.31E-01
MCE	1.00E+00	1.02E+00	2.31E+01	2.36E+01	2.84E+01	3.06E+01
SE	1.40E-02	1.42E-02	6.45E-01	6.49E-01	2.33E+00	2.46E+00

"FDR" indicates the false discovery rate. "MCE" indicates the mean cross-validation error, calculated as binomial deviance for dichotomous traits and mean squared error for quantitative traits, across 10 folds in each replicate. "SE" indicates the standard error of the mean cross-validation error. The power, FDR, number of selected features, λ value, MCE, and SE reported in this table were averaged over 100 replicates for each scenario.

Supplementary Figures

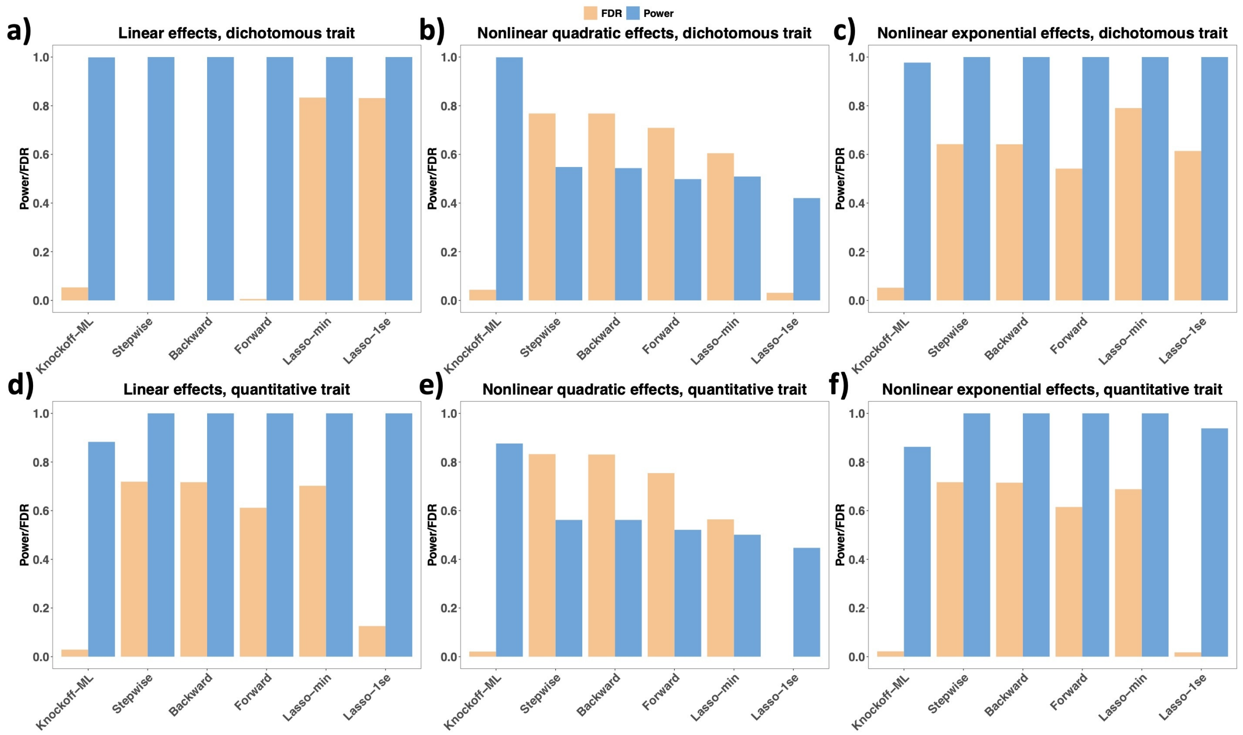

Supplementary Fig. 1: Power and FDR of Knockoff-ML and conventional feature selection methods in simulation studies. The six panels show the power and FDR for different types of traits (**a**, **b**, **c**: dichotomous traits; and **d**, **e**, **f**: quantitative traits) and different types of effects (**a**, **d**: linear effects; **b**, **e**: nonlinear quadratic effects; and **c**, **f**: nonlinear exponential effects). "Knockoff-ML" indicates Knockoff-ML with CatBoost. "Stepwise" indicates stepwise regression. "Backward" indicates backward elimination. "Forward" indicates forward selection. "Lasso-min" indicates lasso with the regularization parameter $\lambda_{\min}$ that gives the minimum mean cross-validation error. "Lasso-1se" indicates lasso with the regularization parameter $\lambda_{1\mathrm{se}}$ that gives the most regularized model such that the cross-validation error is within one standard error of the minimum. The power and FDR for Knockoff-ML were calculated with features identified at a target FDR of 0.1 using CatBoost. CatBoost: categorical boosting; FDR: false discovery rate. The figures were created with the R library ggplot2 and assembled using Microsoft PowerPoint.

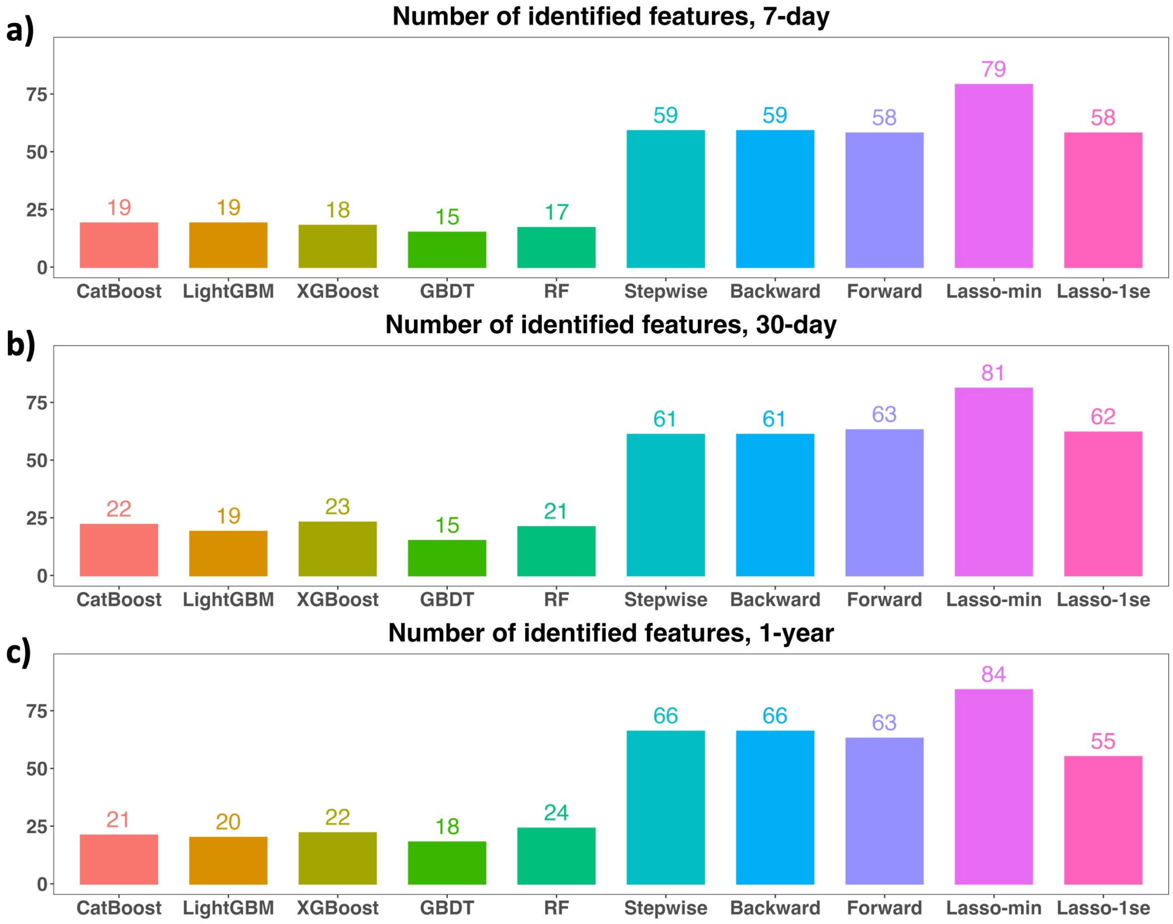

Supplementary Fig. 2: Number of features identified by Knockoff-ML and conventional feature selection methods. The number above each bar represents the number of features identified by the corresponding method on the X-axis. "CatBoost", "LightGBM", "XGBoost", "GBDT", and "RF" indicate Knockoff-ML using the corresponding ML model. "Stepwise" indicates the stepwise regression. "Backward" indicates backward elimination. "Forward" indicates forward selection. "Lasso-min" indicates lasso with the regularization parameter λ_{min} that gives the minimum mean cross-validation error. "Lasso-1se" indicates lasso with the regularization parameter λ_{1se} that gives the most regularized model such that the cross-validation error is within one standard error of the minimum. Outcome of each panel: **a**, 7-day mortality; **b**, 30-day mortality; **c**, 1-year mortality. CatBoost: categorical boosting; LightGBM: light gradient boosting machine; XGBoost: eXtreme gradient boosting; GBDT: gradient boosting decision tree; RF: random forest; Lasso: least absolute shrinkage and selection operator. The figures were created with the R library ggplot2 and assembled using Microsoft PowerPoint.

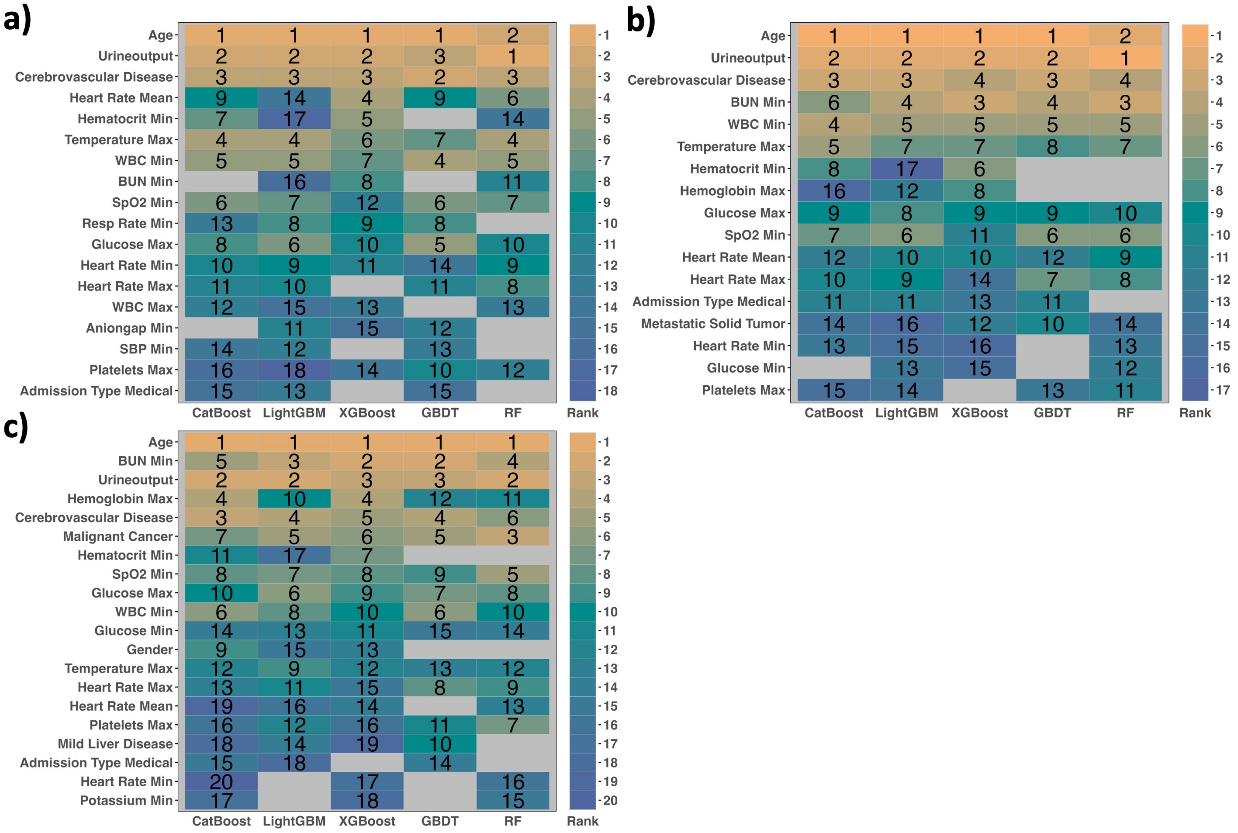

Supplementary Fig. 3: Risk features identified by Knockoff-ML. Features with a select frequency at least three are presented in the figure. The number in each box indicates the ranking of the knockoff statistics W. A gray box without a number indicates that the feature was not selected by Knockoff-ML. Outcomes of each panel: **a**, 7-day mortality; **b**, 30-day mortality; and **c**, 1-year mortality. “Max”, “Mean”, and “Min” indicate the maximum, mean, and minimum values of a risk feature, respectively, within 24 hours after ICU admission. CatBoost: categorical boosting; LightGBM: light gradient boosting machine; XGBoost: eXtreme gradient boosting; GBDT: gradient boosting decision tree; RF: random forest; WBC: white blood cell; BUN: blood urea nitrogen; SBP: systolic blood pressure; Resp Rate: respiratory rate. The figures were created with the R library ggplot2 and assembled using Microsoft PowerPoint.

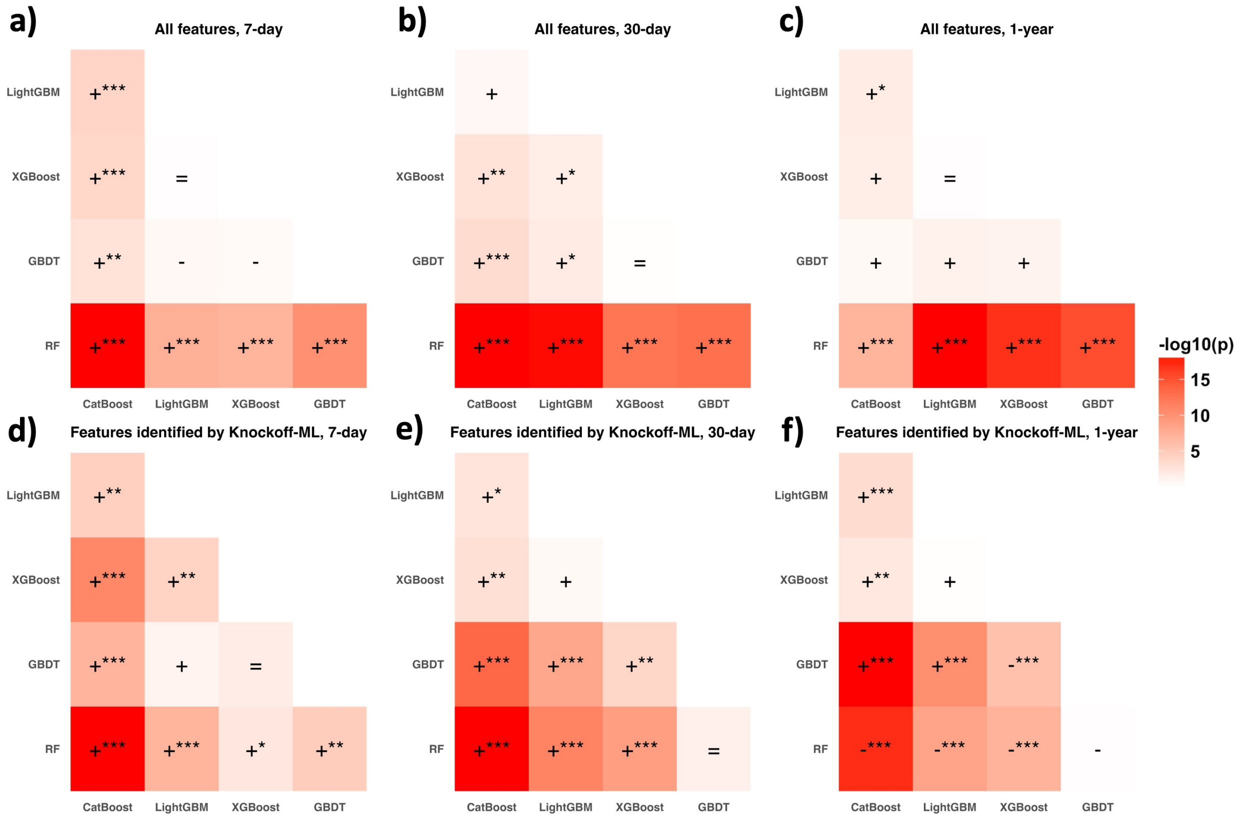

Supplementary Fig. 4: Significance of difference in area under receiver operating characteristic curve (AUROC) between different prediction models. The DeLong test was used to assess whether the difference in AUROCs between two prediction models is statistically significant. "+", "=", and "-" indicate that the AUROCs of models on the X-axis were larger than, equal to, or less than those of the models on the Y-axis, respectively. "***" indicates p-value < 0.001, "**" indicates $0.001 \leq$ p-value < 0.01, "*" indicates $0.01 \leq$ p-value < 0.05, and no star indicates p-value ≥ 0.05. Outcomes of each panel: **a, d**, 7-day mortality; **b, e**, 30-day mortality; **c, f**, 1-year mortality. CatBoost: categorical boosting; LightGBM: light gradient boosting machine; XGBoost: eXtreme gradient boosting; GBDT: gradient boosting decision tree; RF: random forest. The figures were created with the R library ggplot2 and assembled using Microsoft PowerPoint.

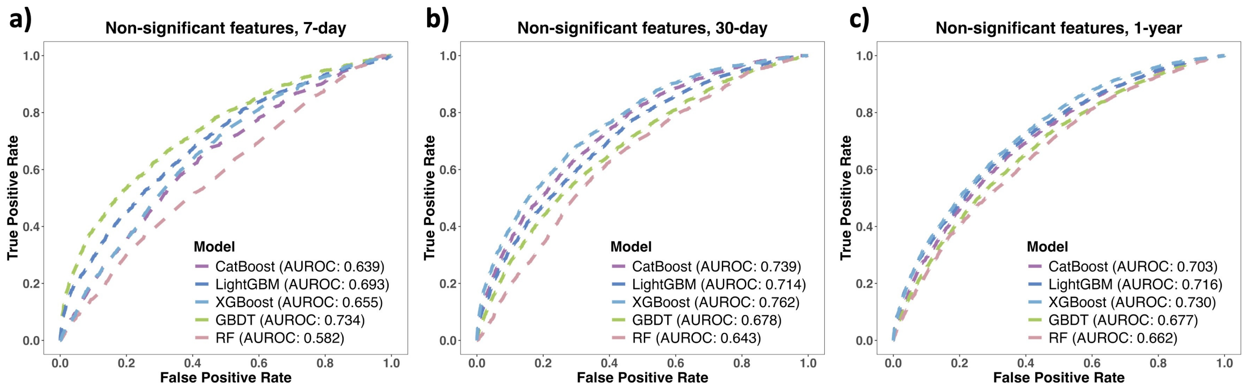

Supplementary Fig. 5: Area under receiver operating characteristic curve (AUROC) for models using Knockoff-ML's lowest-ranked features. The number of lowest-ranked features used in each model was equal to the number of identified features in each model for the corresponding outcome. Outcomes of each panel: **a**, 7-day mortality; **b**, 30-day mortality; **c**, 1-year mortality. CatBoost: categorical boosting; LightGBM: light gradient boosting machine; XGBoost: eXtreme gradient boosting; GBDT: gradient boosting decision tree; RF: random forest. The figures were created with the R library ggplot2 and assembled using Microsoft PowerPoint.

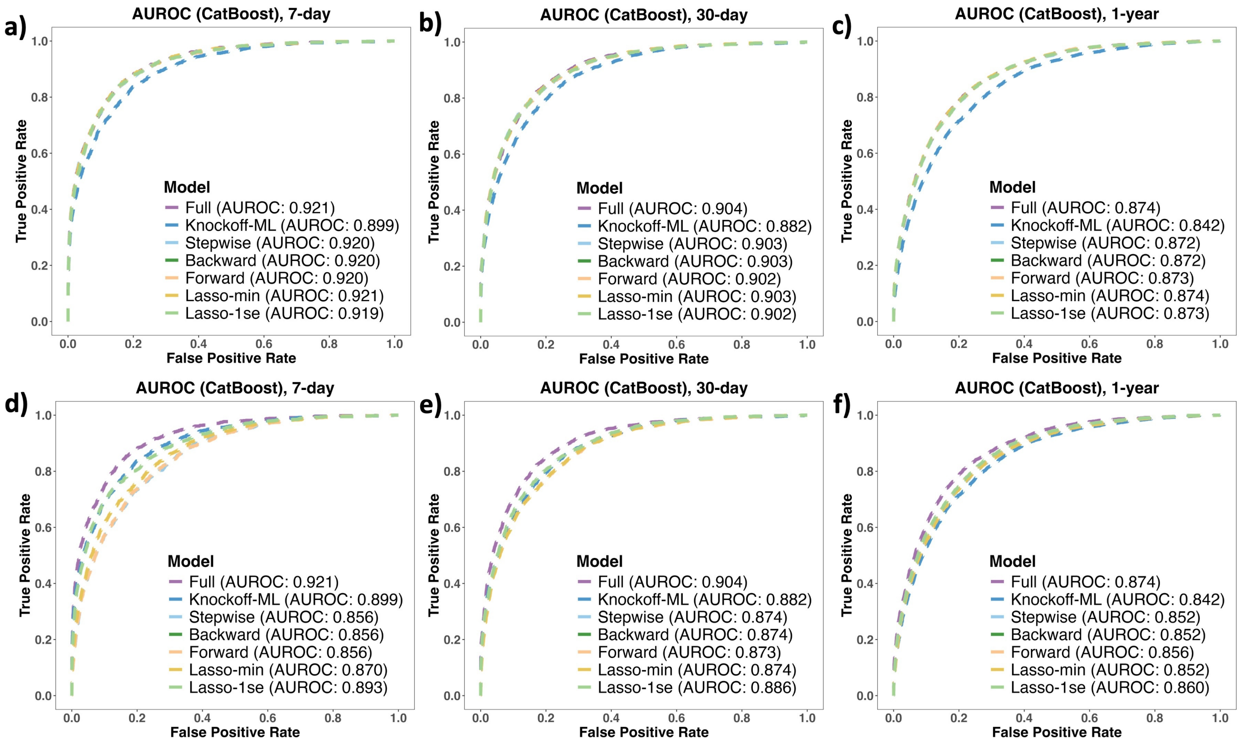

Supplementary Fig. 6: Area under receiver operating characteristic curve (AUROC) for CatBoost using features identified by Knockoff-ML and conventional feature selection methods. CatBoost models were trained with all features and features identified by different feature selection methods, respectively. Outcomes of each panel: **a, d**, 7-day mortality; **b, e**, 30-day mortality; **c, f**, 1-year mortality. In all panels, "Full" indicates models were trained with all available features, "Knockoff-ML" indicates models were trained with features identified by Knockoff-ML using CatBoost, "Stepwise" indicates models were trained with features identified by stepwise regression, "Backward" indicates models were trained with features identified by backward elimination, "Forward" indicates models were trained with features identified by forward selection, "Lasso-min" indicates models were trained with features identified by lasso with the regularization parameter $\lambda_{\min}$ that gives the minimum mean cross-validation error, and "Lasso-1se" indicates models were trained with features identified by lasso with the regularization parameter λ_{1se} that gives the most regularized model such that the cross-validation error is within one standard error of the minimum. In panels **a**, **b**, and **c**, "Stepwise", "Backward", "Forward", "Lasso-min", and "Lasso-1se" trained CatBoost models with all identified features. In panels **d**, **e**, and **f**, "Stepwise", "Backward", "Forward", "Lasso-min", and "Lasso-1se" trained CatBoost models with the highest-ranked identified features based on the absolute values of coefficient estimates $|\hat{\beta}|$, with the number of features set equal to the number of features identified by Knockoff-ML using CatBoost for the corresponding outcome. CatBoost: categorical boosting; lasso: least absolute shrinkage and selection operator. The figures were created with the R library ggplot2 and assembled using Microsoft PowerPoint.

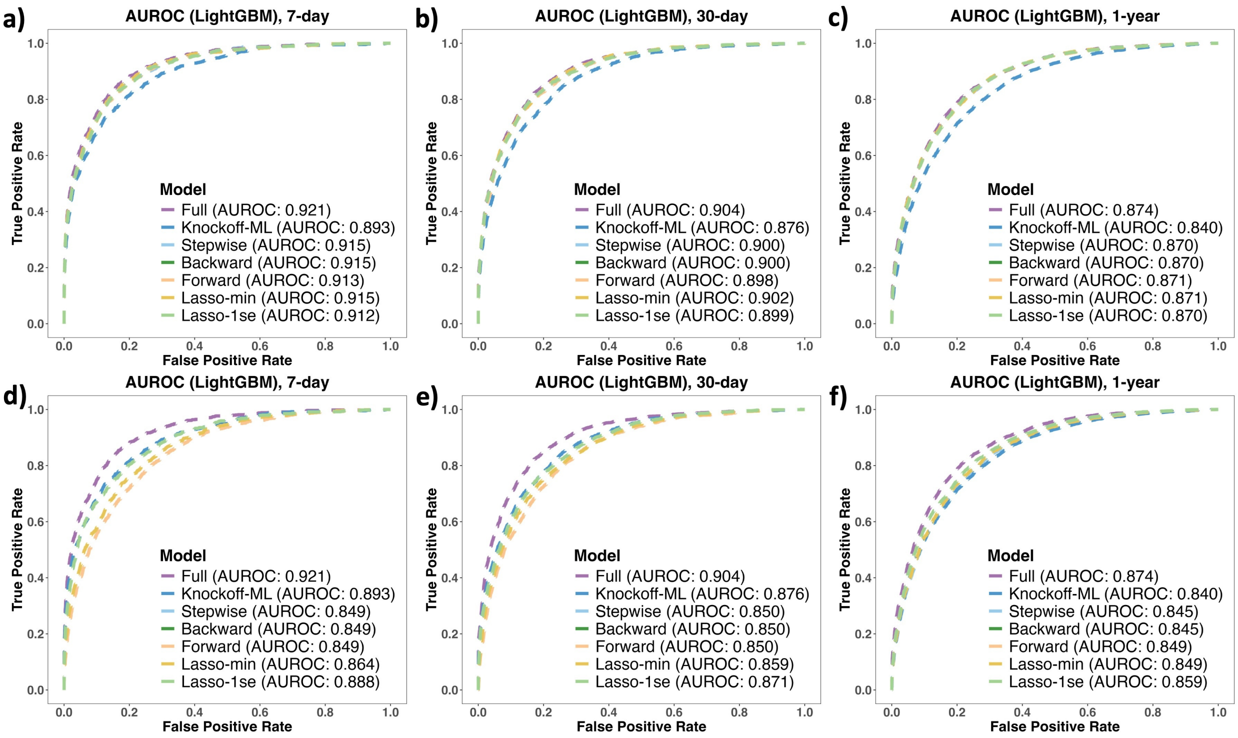

Supplementary Fig. 7: Area under receiver operating characteristic curve (AUROC) for LightGBM using features identified by Knockoff-ML and conventional feature selection methods. LightGBM models were trained with all features and features identified by different feature selection methods, respectively. Outcomes of each panel: **a, d**, 7-day mortality; **b, e**, 30-day mortality; **c, f**, 1-year mortality. In all panels, "Full" indicates models were trained with all available features, "Knockoff-ML" indicates models were trained with features identified by Knockoff-ML using LightGBM, "Stepwise" indicates models were trained with features identified by stepwise regression, "Backward" indicates models were trained with features identified by backward elimination, "Forward" indicates models were trained with features identified by forward selection, "Lasso-min" indicates models were trained with features identified by lasso with the regularization parameter $\lambda_{\min}$ that gives the minimum mean cross-validation error, and "Lasso-1se" indicates models were trained with features identified by lasso with the regularization parameter $\lambda_{1\text{se}}$ that gives the most regularized model such that the cross-validation error is within one standard error of the minimum. In panels **a**, **b**, and **c**, "Stepwise", "Backward", "Forward", "Lasso-min", and "Lasso-1se" trained LightGBM models with all identified features. In panels **d**, **e**, and **f**, "Stepwise", "Backward", "Forward", "Lasso-min", and "Lasso-1se" trained LightGBM models with the highest-ranked identified features based on the absolute values of coefficient estimates $|\hat{\beta}|$, with the number of features set equal to the number of features identified by Knockoff-ML with LightGBM for the corresponding outcome. LightGBM: light gradient boosting machine; lasso: least absolute shrinkage and selection operator. The figures were created with the R library ggplot2 and assembled using Microsoft PowerPoint.

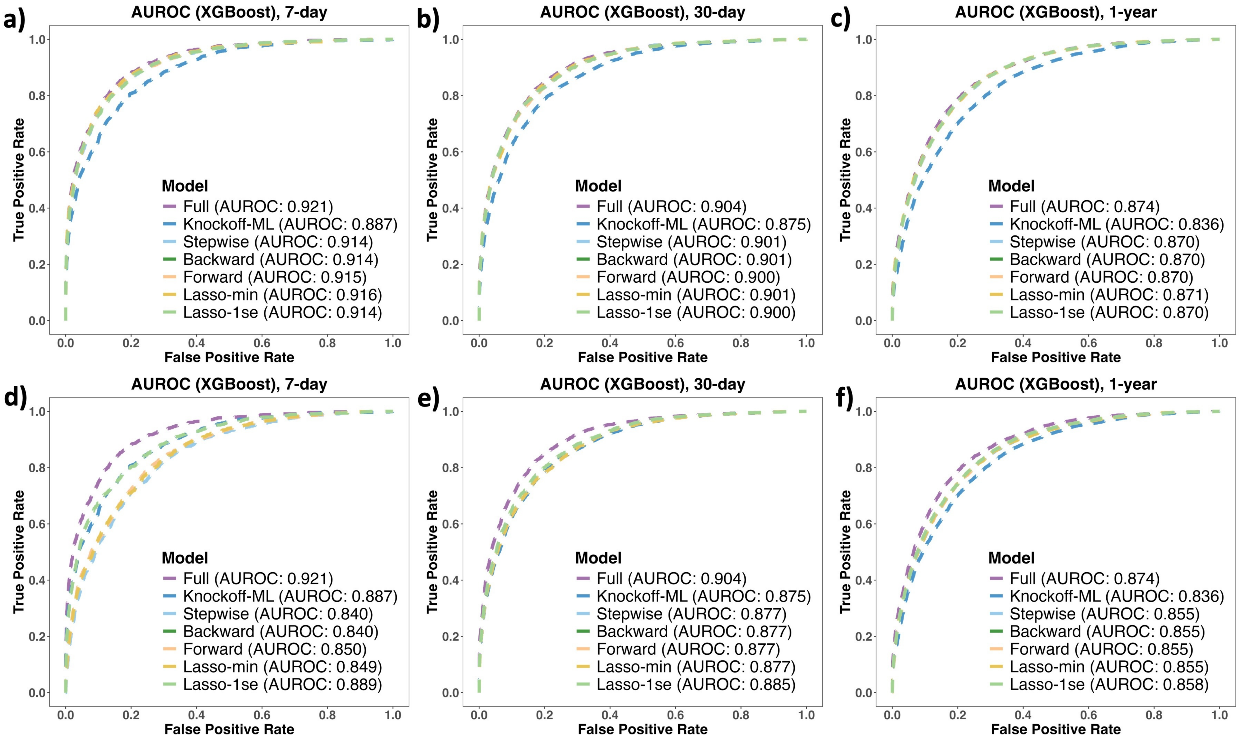

Supplementary Fig. 8: Area under receiver operating characteristic curve (AUROC) for XG-Boost using features identified by Knockoff-ML and conventional feature selection methods. XGBoost models were trained with all features and features identified by different feature selection methods, respectively. Outcomes of each panel: **a, d**, 7-day mortality; **b, e**, 30-day mortality; **c, f**, 1-year mortality. In all panels, "Full" indicates models were trained with all available features, "Knockoff-ML" indicates models were trained with features identified by Knockoff-ML using XGBoost, "Stepwise" indicates models were trained with features identified by stepwise regression, "Backward" indicates models were trained with features identified by backward elimination, "Forward" indicates models were trained with features identified by forward selection, "Lasso-min" indicates models were trained with features identified by lasso with the regularization parameter λ_{min} that gives the minimum mean cross-validation error, and "Lasso-1se" indicates models were trained with features identified by lasso with the regularization parameter λ_{1se} that gives the most regularized model such that the cross-validation error is within one standard error of the minimum. In panels **a**, **b**, and **c**, "Stepwise", "Backward", "Forward", "Lasso-min", and "Lasso-1se" trained XGBoost models with all identified features. In panels **d**, **e**, and **f**, "Stepwise", "Backward", "Forward", "Lasso-min", and "Lasso-1se" trained XGBoost models with the highest-ranked identified features based on the absolute values of coefficient estimates $|\hat{\beta}|$, with the number of features set equal to the number of features identified by Knockoff-ML with XGBoost for the corresponding outcome. XGBoost: eXtreme gradient boosting; lasso: least absolute shrinkage and selection operator. The figures were created with the R library ggplot2 and assembled using Microsoft PowerPoint.

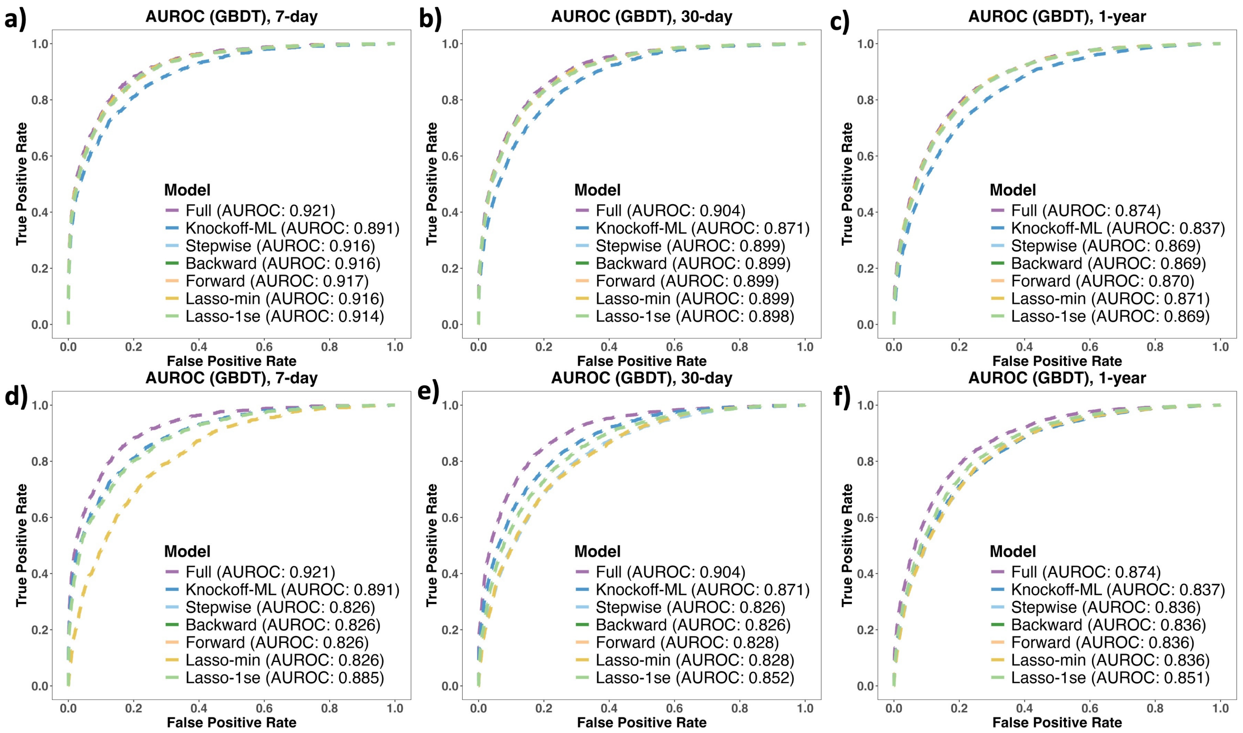

Supplementary Fig. 9: Area under receiver operating characteristic curve (AUROC) for GBDT using features identified by Knockoff-ML and conventional feature selection methods. GBDT models were trained with all features and features identified by different feature selection methods, respectively. Outcomes of each panel: **a, d**, 7-day mortality; **b, e**, 30-day mortality; **c, f**, 1-year mortality. In all panels, "Full" indicates models were trained with all available features, "Knockoff-ML" indicates models were trained with features identified by Knockoff-ML using GBDT, "Stepwise" indicates models were trained with features identified by stepwise regression, "Backward" indicates models were trained with features identified by backward elimination, "Forward" indicates models were trained with features identified by forward selection, "Lasso-min" indicates models were trained with features identified by lasso with the regularization parameter $\lambda_{\min}$ that gives the minimum mean cross-validation error, and "Lasso-1se" indicates models were trained with features identified by lasso with the regularization parameter λ_{1se} that gives the most regularized model such that the cross-validation error is within one standard error of the minimum. In panels **a**, **b**, and **c**, "Stepwise", "Backward", "Forward", "Lasso-min", and "Lasso-1se" trained GBDT models with all identified features. In panels **d**, **e**, and **f**, "Stepwise", "Backward", "Forward", "Lasso-min", and "Lasso-1se" trained GBDT models with the highest-ranked identified features based on the absolute values of coefficient estimates $|\hat{\beta}|$, with the number of features set equal to the number of features identified by Knockoff-ML with GBDT for the corresponding outcome. GBDT: gradient boosting decision tree; lasso: least absolute shrinkage and selection operator. The figures were created with the R library ggplot2 and assembled using Microsoft PowerPoint.

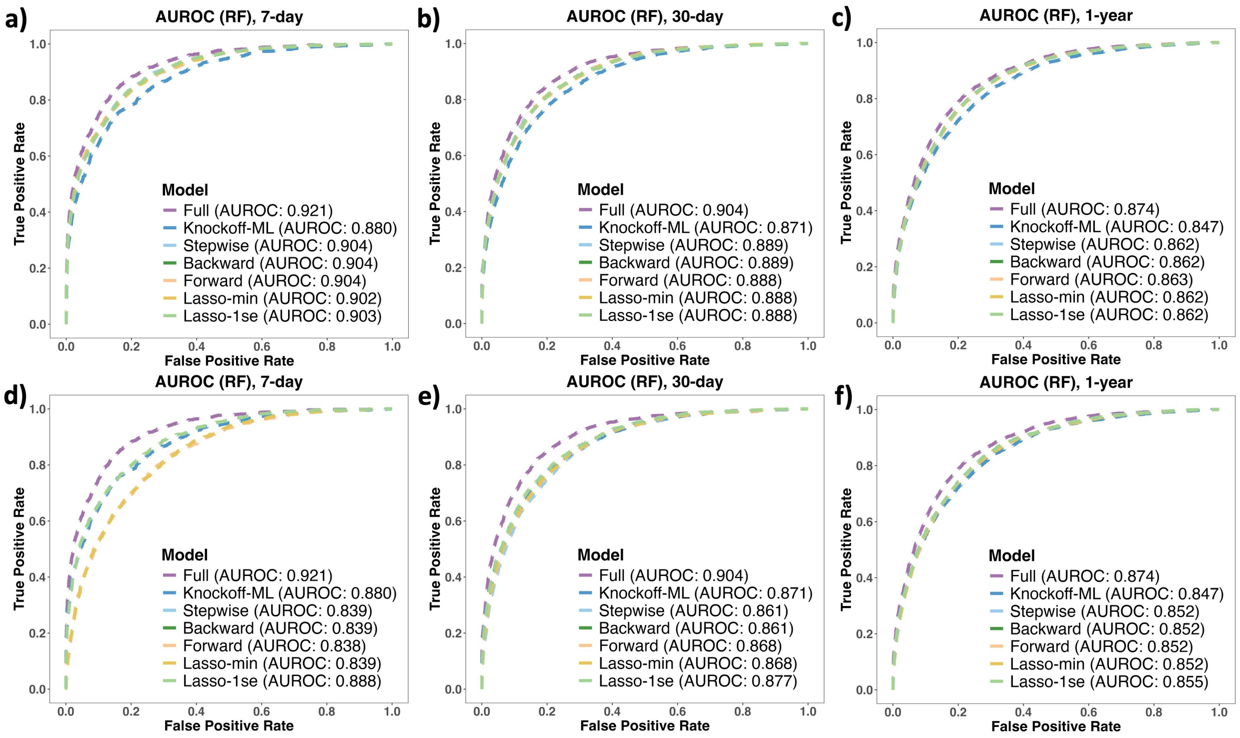

Supplementary Fig. 10: Area under receiver operating characteristic curve (AUROC) for RF using features identified by Knockoff-ML and conventional feature selection methods. RF models were trained with all features and features identified by different feature selection methods, respectively. Outcomes of each panel: **a, d**, 7-day mortality; **b, e**, 30-day mortality; **c, f**, 1-year mortality. In all panels, "Full" indicates models were trained with all available features, "Knockoff-ML" indicates models were trained with features identified by Knockoff-ML using RF, "Stepwise" indicates models were trained with features identified by stepwise regression, "Backward" indicates models were trained with features identified by backward elimination, "Forward" indicates models were trained with features identified by forward selection, "Lasso-min" indicates models were trained with features identified by lasso with the regularization parameter λ_{min} that gives the minimum mean cross-validation error, and "Lasso-1se" indicates models were trained with features identified by lasso with the regularization parameter λ_{1se} that gives the most regularized model such that the cross-validation error is within one standard error of the minimum. In panels **a**, **b**, and **c**, "Stepwise", "Backward", "Forward", "Lasso-min", and "Lasso-1se" trained RF models with all identified features. In panels **d**, **e**, and **f**, "Stepwise", "Backward", "Forward", "Lasso-min", and "Lasso-1se" trained RF models with the highest-ranked identified features based on the absolute values of coefficient estimates $|\hat{\beta}|$, with the number of features set equal to the number of features identified by Knockoff-ML with RF for the corresponding outcome. RF: random forest; lasso: least absolute shrinkage and selection operator. The figures were created with the R library ggplot2 and assembled using Microsoft PowerPoint.

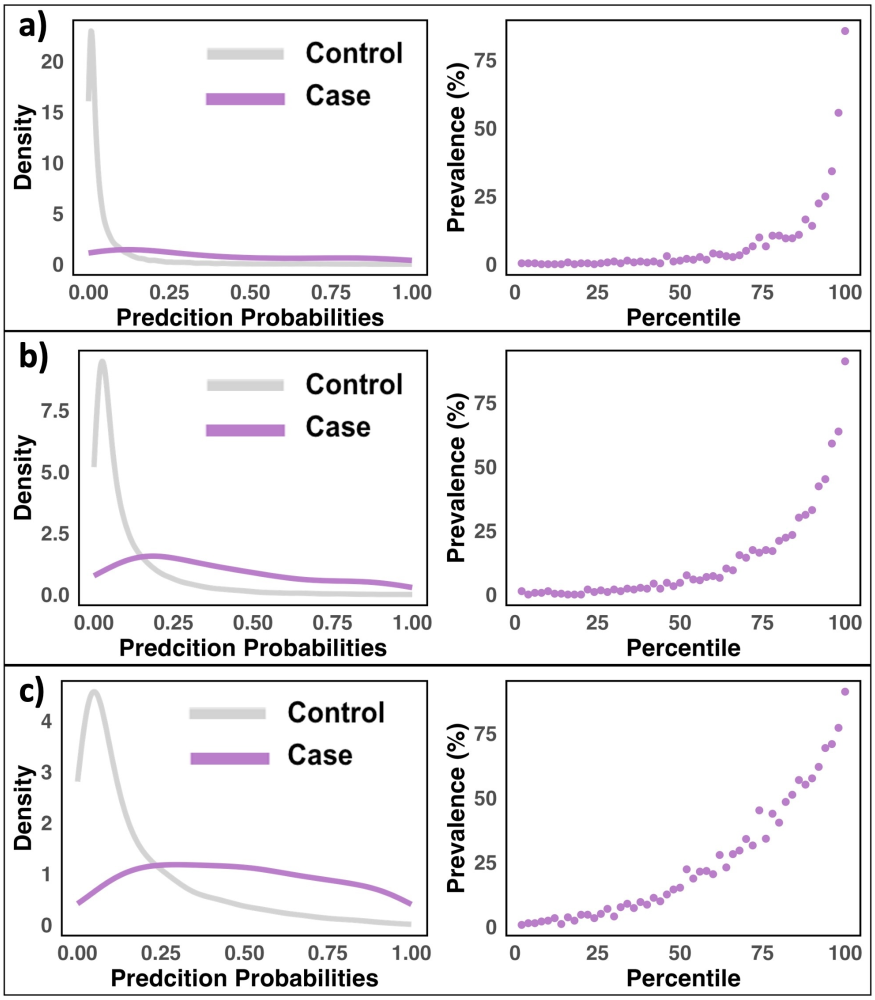

Supplementary Fig. 11: Distribution of predicted probabilities from final prediction models based on risk features identified by Knockoff-ML for mortality outcomes. For each outcome: left, estimated density of the prediction probabilities of cases and controls, respectively, in the test set; right, case prevalence in 50 bins according to the percentiles of prediction probabilities. Outcomes of each panel: **a**, 7-day mortality; **b**, 30-day mortality; **c**, 1-year mortality. The figures were created with the R library ggplot2 and assembled using Microsoft PowerPoint.

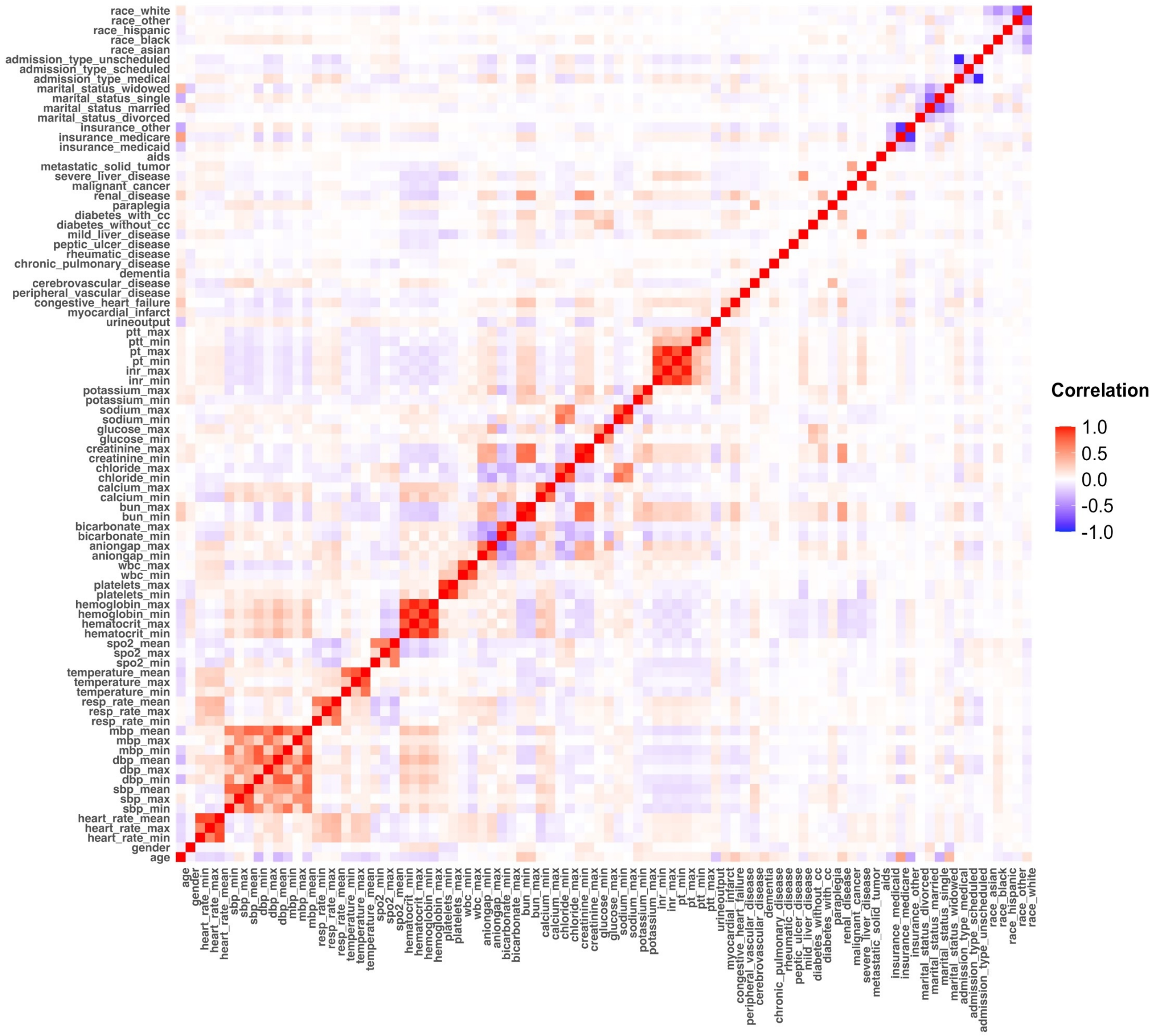

Supplementary Fig. 12: Heatmap of feature correlations in the MIMIC-IV database. Pearson's correlation coefficients between features are shown as colors ranging from blue (negative correlation) to red (positive correlation), with white indicating no correlation. "Max", "mean", and "min" indicate the maximum, mean, and minimum values of a vital sign or lab test. The figure was created with the R library ggplot2.

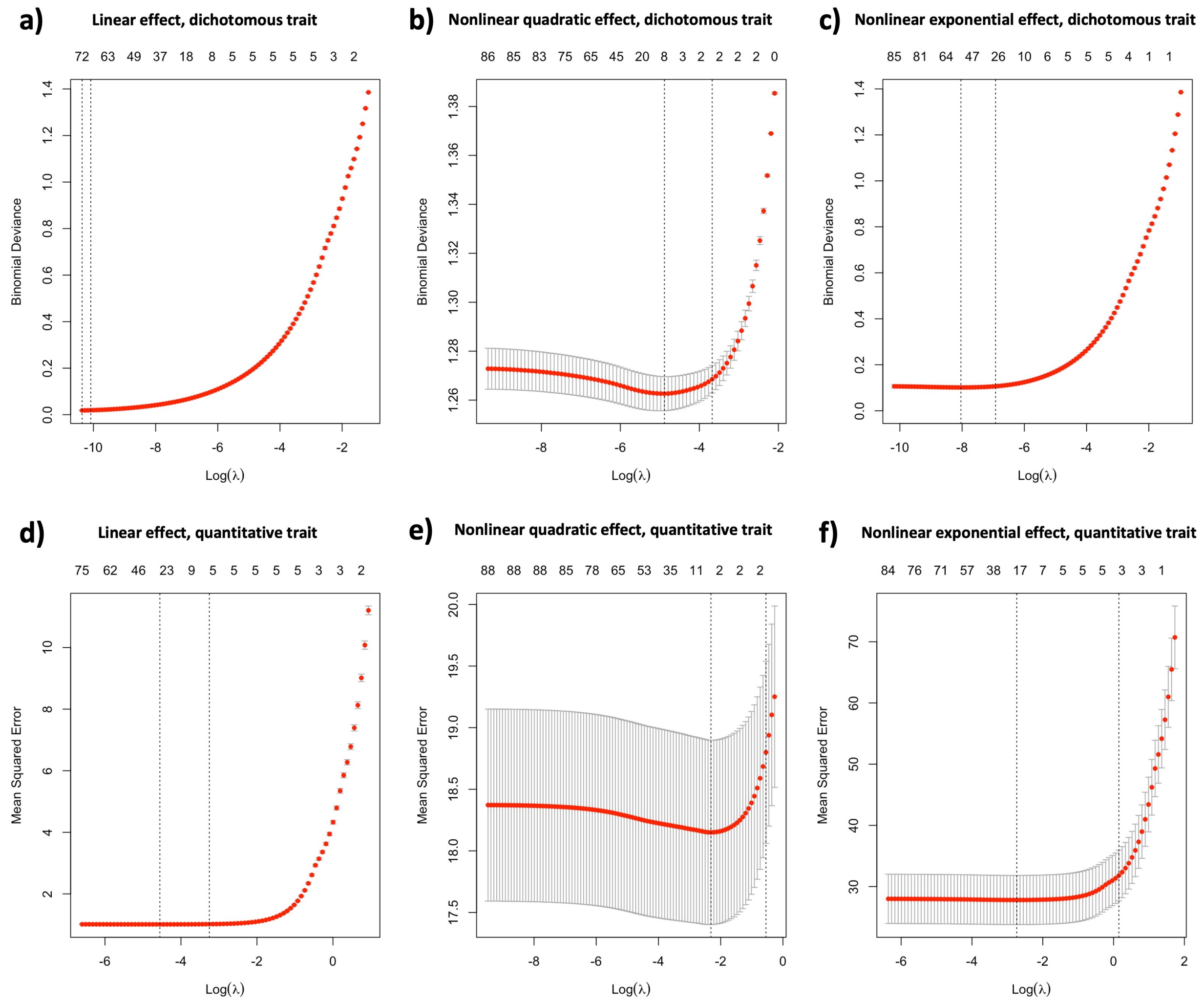

Supplementary Fig. 13: Mean cross-validation error in lasso with different λ values. The six panels show mean cross-validation error in lasso evaluated at different λ values for different types of traits (**a**, **b**, **c**: dichotomous traits, and **d**, **e**, **f**: quantitative traits) and different types of effects (**a**, **d**: linear effects, **b**, **e**: nonlinear quadratic effects, and **c**, **f**: nonlinear exponential effects) in a randomly selected replicate. The X-axis indicates the natural logarithm of λ. The Y-axis indicates the mean cross-validation error calculated as binomial deviance for dichotomous traits and mean squared error for quantitative traits across 10 folds. In each panel, the dotted vertical line on the left represents $\lambda_{\min}$, and the dotted vertical line on the right represents λ_{1se}. The figure was created with the R function plot and assembled using Microsoft PowerPoint.
